# Spermidine improves seed viability in *Allium mongolicum* by regulating AmCS-mediated metabolic and antioxidant networks

**DOI:** 10.3389/fpls.2025.1683362

**Published:** 2025-10-08

**Authors:** Yan Men, Pengchao Wei, Fenglan Zhang, Xiumei Huang, Dong Zhang, Rongji Feng, Xueqin He, Zhongren Yang

**Affiliations:** College of Horticulture and Plant Protection, Inner Mongolia Agricultural University, Hohhot, China

**Keywords:** *Allium mongolicum*, spermidine priming, citrate synthase, energy metabolism, ROS scavenging, overexpression

## Abstract

**Introduction:**

Seed deterioration involves oxidative damage and disrupted energy metabolism, yet the genetic mechanisms underlying aging resistance in *Allium mongolicum* remain unclear.

**Methods:**

In this study, seeds primed with 0.8 mM spermidine (Spd) and stored for varying durations were subjected to transcriptome sequencing, targeted energy metabolite profiling, and assessments of antioxidant systems and energy metabolism enzymes.

**Results:**

We identified citrate synthase (*AmCS*) as a pivotal candidate gene involved in delaying aging processes. Under standard growth conditions, *AmCS*-overexpressing *Arabidopsis* lines exhibited a 15.55% higher germination rate compared to wild-type (WT), with enhanced activities of superoxide dismutase (SOD) and peroxidase (POD), and a 46.37% increase in ATP content compared to WT. Furthermore, these transgenic lines displayed significant reductions in hydrogen peroxide (H_2_O_2_; 35.20%) and malondialdehyde (MDA; 40.40%) accumulation. Mechanistically, *AmCS*-overexpressing *Arabidopsis* lines demonstrated heightened mitochondrial functionality, manifested as a 50.26% increase in cellular respiration rate and a 1.41-fold higher NADPH/NADP^+^ ratio than WT. Yeast two-hybrid assays validated the physical interaction between AmCS and pyruvate dehydrogenase kinase (AmPDK).

**Discussion:**

We demonstrate that the AmCS-AmPDK complex retards seed aging through two key mechanisms: (i) promoting acetyl-CoA flux in the tricarboxylic acid (TCA) cycle and (ii) enhancing NADPH-dependent antioxidant capacity through pentose phosphate pathway activation. Exogenous Spd activates this network by inducing *AmCS* expression. Our findings establish *AmCS* as a key genetic regulator for enhancing anti-aging traits in crop breeding, offering prospects for precision breeding and advancements in seed storage practices.

## 1 Introduction

Seed germination is a crucial developmental stage in plant life cycles, with significant implications for *ex situ* conservation and agricultural productivity. The longevity of seeds, as essential genetic repositories, is governed by a complex interplay of genetic, physiological, and storage-related factors (Pereira-Lima et al., 2017). Post-maturation seed vigor undergoes progressive decline during storage (Popov et al., 2022), attributed to a molecular damage cascade involving the breakdown of antioxidant systems (Kranner et al., 2010), disturbances in energy metabolism, membrane lipid peroxidation, and macromolecular deterioration (Zinsmeister et al., 2020). These cumulative damages ultimately manifest as reduced germination rates and abnormal seedling phenotypes (Hang and Mao, 2007). Recent studies have highlighted the involvement of epigenetic regulation in aging processes (Zhang et al., 2024). However, the key genetic drivers and underlying molecular mechanisms in non-model crops like *Allium mongolicum* remain poorly understood. The lack of genomic resources and efficient transformation protocols in these crops exacerbate this knowledge gap, hindering the development of delayed-aging strategies.

Polyamines, a class of essential physiological regulators in plants, serve dual functions in tissue development and maintenance of cellular membrane integrity. Among these, spermidine (Spd), a representative polyamine, plays a crucial role in various physiological processes such as photosynthesis, metabolic regulation, respiration, organogenesis, and stress responses (Bhukel et al., 2017; Fu et al., 2019). Under abiotic stress conditions, Spd enhances plant antioxidant capacity by reducing the accumulation of reactive oxygen species (ROS, e.g., hydrogen peroxide, H_2_O_2_) and lipid peroxidation markers (e.g., malondialdehyde, MDA), thereby alleviating stress-induced damage (Huang et al., 2023; Ma, 2023; Sawariya et al., 2024). Notably, the profiles of polyamines, particularly Spd, are strongly associated with seed germination capacity, with reduced Spd levels accelerating seed aging processes (Hu et al., 2020; Lerin et al., 2022). In aged sorghum seeds, Spd priming enhances carbohydrate metabolism-related enzyme activities, improves germination efficiency, and reduces ROS accumulation (Zhang et al., 2023). Similarly, studies in rice demonstrate that exogenous Spd treatment upregulates both antioxidant enzyme activities and their corresponding genes. Conversely, inhibition of Spd biosynthesis significantly reduces germination rates and impairs seedling establishment (Fu et al., 2019). These findings highlight the role of Spd in coordinating a synergistic network that connects energy metabolism with oxidative defense systems, thereby counteracting the inhibition of germination induced by aging. This understanding of the underlying mechanisms offers potential targets, such as key enzymes or genes, for rejuvenating aged seeds. Nevertheless, the regulatory mechanisms governing Spd function in specialty crops such as *Allium mongolicum* remain unclear, presenting a significant gap in seed longevity research.

Besides antioxidant system impairment, energy supply disruption is another characteristic feature of seed aging. Adenosine triphosphate (ATP), the universal energy currency, drives a wide range of physiological processes crucial for plant growth, defense responses, respiration, and metabolic functions. As the direct energy carrier for seed germination and seedling establishment, ATP production relies on three interconnected pathways: glycolysis, tricarboxylic acid (TCA) cycle and the pentose phosphate pathway (PPP), collectively constituting the central energy metabolism network (Qu et al., 2020). Glycolysis initiates energy production by converting glucose into pyruvate and ATP, while the TCA cycle, the core of aerobic respiration, fully oxidizes pyruvate to generate NADH and FADH_2_ for mitochondrial ATP synthesis. Concurrently, the PPP produces NADPH and ribose-5-phosphate, contributing to the maintenance of redox homeostasis and nucleotide biosynthesis (Bewley et al., 2013; Eastmond and Graham, 2001). During seed aging, the activities of key glycolytic enzymes (e.g., hexokinase and pyruvate kinase) are significantly reduced, leading to ATP deficiency and consequent plasma membrane disintegration (Bailly et al., 2008). TCA cycle dysfunction is characterized by diminished activities of citrate synthase (CS) and isocitrate dehydrogenase, resulting in a shortage of acetyl-CoA and deterioration of mitochondrial function (Bhatti et al., 2017). PPP impairment, by limiting NADPH supply, exacerbates ROS accumulation, establishing a vicious cycle between metabolic breakdown and oxidative stress (Zhou et al., 2025). Notably, polyamines like Spd have the capacity to concurrently rectify energy-oxidative imbalances by reinstating the coupling between glycolysis and respiration (Zhang et al., 2023), enhancing TCA cycle flux (Fu et al., 2019), and upregulating PPP rate-limiting enzymes, such as glucose-6-phosphate dehydrogenase (G6PDH, Ma, 2023). Nevertheless, the precise molecular mechanisms through which Spd influences the activity of the TCA cycle remain obscure, especially in non-model species with exceptional desiccation tolerance such as *Allium mongolicum*.

*Allium mongolicum* Regel (Amaryllidaceae), a perennial xerophytic herb native to arid Central Asia, is a keystone species in Gobi desert ecosystems renowned for its robust root system and exceptional resilience to various stresses (drought, cold, and pathogens). Its significant role in maintaining ecological functions in desert environments underscores its importance (Zhang et al., 2024; Wang et al., 2013). The remarkable adaptability of *A. mongolicum* to extreme conditions positions it as an excellent model for investigating mechanisms of aging resistance in plants. In natural desert vegetation regeneration, seed propagation is fundamental for sustaining populations, yet seed aging presents a substantial obstacle to population renewal, with its molecular regulation largely unexplored. While Spd has demonstrated anti-aging effects through enhancing antioxidant capacity and energy metabolism (Zhang et al., 2023), and citrate synthase (CS)—the rate-limiting enzyme of the TCA cycle—has been linked to seed vigor in crops like sunflower (Cai et al., 2022), critical gaps in knowledge persist. Recent research indicates that CS may delay aging by maintaining mitochondrial membrane potential and reducing ROS accumulation (Zhang et al., 2023), while Spd has been shown to epigenetically regulate the activities of enzymes in the TCA cycle (Fu et al., 2019). However, their synergistic mechanisms in *A. mongolicum* seeds have not been investigated. This study aims to investigate the anti-aging mechanism of CS-mediated metabolic networks in naturally aged *A. mongolicum* seeds treated with exogenous Spd, employing RNA-seq transcriptomics, targeted metabolomics, and heterologous expression in *Arabidopsis*, as well as to quantitatively evaluate the restorative effects of *CS* overexpression on aged seeds. Our findings will not only identify *CS* as a key regulator of seed aging resistance in desert plants but also offer potential molecular targets for seed vigor enhancers based on *CS*, establishing a novel xerophyte model for aging research.

## 2 Materials and methods

### 2.1 Plant materials and experimental treatments

*Allium mongolicum* seeds were collected in October 2019, 2021, and 2023 from natural habitats in Otog Front Banner, Inner Mongolia, China (38°18’N, 107°29’E; altitude 1,320 m), characterized by Arenosols according to FAO soil classification, with an annual precipitation of 280 mm (data from the Grassland Ecosystem Research Station, Chinese Academy of Sciences, 2024). Following collection, mature seeds from 50 randomly selected healthy individuals were air-dried (25 ± 1 °C, 30% relative humidity) and stored in gas-tight aluminum foil bags containing silica gel desiccant (5 g per kg seeds) at 4 °C (maintaining 15 ± 3% relative humidity, RH). The study involved three storage durations: freshly harvested seeds (0-year storage, collected in 2023), seeds stored for 2 years (collected in 2021), and seeds stored for 4 years (collected in 2019). The treatment group (TR) underwent a 12 h imbibition in a 0.8 mM Spd aqueous solution at 25 ± 1 °C, while the control group (CK) was imbibed an equal volume of double-distilled water under identical conditions (Men et al., 2025). Treated seeds were placed on sterilized filter paper in 9-cm Petri dishes with 5 mL of the treatment solution per dish and incubated in climate chambers (RXZ-500D, Ningbo, China) under controlled conditions (20 ± 1 °C, 90% RH, darkness). The solution volume was maintained constant by daily replenishment guided by weight measurements. Germination was defined as radicle emergence ≥1 mm and recorded for calculating germination rate. Samples of treated seeds were promptly frozen in liquid nitrogen and stored at -80 °C (Thermo Scientific) in RNase-free 5 mL tubes (Axygen). All physiological and biochemical analyses were completed within 30 days of sample collection. The experimental design adhered to a completely randomized layout encompassing six treatment combinations (3 storage durations × 2 imbibition treatments), with each treatment comprising three biological replicates (consisting of 100 seeds per replicate).

### 2.2 Determination of key metabolic enzyme activities and ATP content

Key enzyme activities and ATP levels were assessed utilizing microplate reader assays (BioTek Synergy H1, Winooski, VT, USA) and UV spectrophotometric assays (Shimadzu UV-2600, Kyoto, Japan). Enzyme activities were quantified as follows: Isocitrate dehydrogenase (IDH; Kit No. BC2165), Citrate synthase (CS; Kit No. BC1065), Malate dehydrogenase (MDH; Kit No. BC1045), Hexokinase (HK; Kit No. BC0745), α-Ketoglutarate dehydrogenase (α-OGDH; Kit No. BC0170), Pyruvate kinase (PK; Kit No. BC0540), Phosphofructokinase (PFK; Kit No. BC0530), Glucose-6-phosphate dehydrogenase (G6PDH; Kit No. BC0260), and β-galactosidase (β-gal; Kit No. BC2585). The ATP content was determined using Kit No. BC0300. Standard curves ranging from 0*–*100 μmol·L⁻¹ at 25 ± 1 °C were constructed, incorporating enzyme-free (heat-inactivated extracts) and substrate-free controls. All activities were expressed as specific activity (U mg⁻¹ protein), with protein concentrations determined by BCA assay (Kit No. PC0020) at 562 nm, employing bovine serum albumin (BSA, Sigma-Aldrich Cohn Fraction V) as standard. Three independent biological replicates were analyzed. All kits were purchased from Solarbio Life Sciences (Beijing, China).

### 2.3 Transcriptome sequencing and analysis

Total RNA was extracted from seeds using the modified CTAB method (Liang et al., 2016), followed by dissolution in 50 µL of diethyl pyrocarbonate (DEPC)-treated water. RNA concentration was quantified using a Qubit 4.0 fluorometer (Thermo Fisher Scientific, Waltham, MA, USA), and RNA integrity number (RIN) > 7.0 was assessed with a Qsep400 Bio-Fragment Analyzer (BiOptic Inc., Taiwan, China). Subsequent to quality control, mRNA was isolated via poly(A) selection, and cDNA libraries were prepared following standard Illumina protocols. These steps included mRNA fragmentation (200–300 bp), first- and second-strand cDNA synthesis, end repair, A-tailing, adapter ligation, AMPure XP bead (Beckman Coulter, Brea, CA, USA) purification, PCR amplification, and circularization. The resulting libraries were quantified using Qubit 4.0, and insert fragment size distribution was confirmed using an Agilent 2100 Bioanalyzer (Agilent Technologies, Santa Clara, CA, USA). Prior to DNBSEQ sequencing, the libraries were circularized and amplified via rolling circle amplification (RCA) using phi29 DNA polymerase to generate DNA nanoballs (DNBs), each containing >300 copies of the DNA molecule. These DNBs were loaded onto sequencing chips, and 150-bp paired-end sequencing was carried out on the DNBSEQ platform (Metware Biotechnology Co., Ltd., Wuhan, China). The raw reads were quality-filtered with fastp (v0.23.2) by discarding sequences containing adapters, ambiguous bases (>5%), or low-quality scores (Q20<). Cleaned reads were assembled *de novo* using Trinity (v2.15.1; k-mer=25, min_contig_length=200). Transcript clusters were generated using Corset v1.09 to reduce redundancy (Davidson and Oshlack, 2014). Functional annotation of transcripts involved aligning sequences to seven databases: NR, Swiss-Prot, KEGG, GO, KOG (Tatusov et al., 2000), Pfam, and TrEMBL using Diamond v2.0.9 (Buchfink et al., 2015), while protein domain prediction was executed via HMMER v3.3.2 against the Pfam database (v35.0). Gene expression levels were quantified using RSEM (v1.3.3) (Li and Dewey, 2011; Langmead and Salzberg, 2012), with expression levels reported in FPKM. Differential expression analysis was performed with DESeq2 (v1.38.3) (Love et al., 2014) using a significance threshold of |log2(fold change)| ≥ 2 and FDR-adjusted p-value < 0.05 (Benjamini-Hochberg method). Functional characterization included separate enrichment analyses for KEGG pathways (Kanehisa et al., 2008) and Gene Ontology (GO) terms (Ashburner et al., 2000) using the hypergeometric test. Three biological replicates were analyzed per condition.

### 2.4 Targeted metabolomic profiling of energy metabolism

For targeted metabolomic analysis of energy metabolism pathways, frozen samples were thawed and homogenized before aliquots (50 mg) were extracted with a biphasic solvent system containing 500 μL of methanol/water (50:50, v/v) (with isotope-labeled internal standards) and 500 μL dichloromethane. After vigorous mixing (2500 × g, 3 min) and centrifugation (12,000 × g, 4 °C, 10 min), 300 μL of the upper methanol/water phase was collected, chilled at -20 °C for 30 min, and recentrifuged under identical conditions. The resulting extract (200 μL) was filtered through a protein precipitation plate prior to LC-MS analysis using an ACQUITY H-Class UPLC system (Waters) coupled to a QTRAP 6500^+^ mass spectrometer (Sciex) (Guo et al., 2013). Chromatographic separation was achieved on an ACQUITY UPLC BEH Amide column (2.1 × 100 mm, 1.7 μm) with mobile phase A (10 mM ammonium acetate and 0.3% (v/v) ammonium hydroxide in water) and B (acetonitrile/water, 90:10, v/v) using a gradient program: 95% B (0*–*1.2 min), linear decrease to 70% B (8 min), step transition to 50% B at 8 min (maintained until 11 min), followed by re-equilibration at 95% B (11.1–15 min) at 0.4 mL/min flow rate and 40 °C column temperature with 2 μL injection volume. Mass spectrometric detection was performed in both positive and negative ionization modes (ion spray voltage: +5500 V, positive mode; -4500 V, negative mode) with source temperature at 550 °C and curtain gas at 35 psi, employing scheduled multiple reaction monitoring (MRM) with optimized declustering potentials and collision energies (Luo et al., 2007; Cordell et al., 2008; Gil et al., 2015). Data acquisition used Analyst 1.6.3 software while quantification was performed with MultiQuant 3.0.3, followed by statistical analysis in MetaboAnalystR using VIP scores and absolute log_2_ fold-change values (|log_2_FC|) for significance determination (Lorenzetti et al., 2007; Chong and Xia, 2018; An et al., 2020). All analyses were conducted by Wuhan Metware Biotechnology Co., Ltd. using the QTRAP 6500^+^ platform.

### 2.5 Functional analysis of the citrate synthase gene (*AmCS*)

#### 2.5.1 Construction of the *AmCS* overexpression vector and bioinformatics analysis

In our transcriptome analysis, the citrate synthase gene (*AmCS*) from *Allium mongolicum* was identified as a promising candidate for anti-aging studies. Primers were designed using Primer 5.0 (**Supplementary Table S1**; **Supplementary File 1**). Total RNA extraction was performed with the FastPure Universal Plant Total RNA Isolation Kit (Vazyme, RC411), and concentration/purity (A260/A280 ratio: 1.8*–*2.1) were verified by Nanodrop ND-1000. First-strand cDNA synthesis was carried out using the HiScript II 1st Strand cDNA Synthesis Kit (Vazyme, R212), diluted to 200 ng·μL⁻¹. *AmCS* gene was amplified by PCR (Max Super-Fidelity DNA Polymerase, Vazyme P505-D1; **Supplementary Table S2**), visualized on 1% agarose gel (120 V, 30 min) with DL2000 DNA Marker, gel-purified (TIANGEN DP209-03), and column-purified (GenStar D205-01). Purified fragment and pEGOE35S vector digested with EcoRI-HF/HindIII-HF (NEB) at 37 °C, ligated with T4 DNA Ligase (TaKaRa 6022A), and transformed into DH5α competent cells (TIANGEN CB101). Positive clones selected on hygromycin (50 mg/L) plates, validated by Sanger sequencing (T7 primer, **Supplementary Table S1**). Bioinformatics analysis followed Sun et al. (2024): Sequence translation (DNAMAN); Physicochemical properties (ExPASy ProtParam); Transmembrane domains (TMHMM v2.0); Signal peptides (SignalP v5.0, eukaryotic); Secondary structure (SOPMA); Tertiary structure (SWISS-MODEL template: A. thaliana Q9C932); Phosphorylation sites (NetPhos v3.1, threshold>0.5); N-glycosylation sites (NetNGlyc 1.0). Phylogenetic tree was constructed in MEGA v11.0 (Neighbor-joining, Poisson correction model with Gamma-distributed rates, 1000 bootstraps). All analyses used default parameters.

#### 2.5.2 Heterologous overexpression of the *AmCS* gene

*AmCS* was expressed in *Arabidopsis thaliana* (Col-0) via floral dip using *Agrobacterium tumefaciens* GV3101 (Sangon Biotech, A339088). Healthy 4-5-week-old plants grown under long-day conditions (16 h light/8 h dark, 22 ± 1 °C, 100 ± 5 μmol·m⁻²·s*⁻¹) with primary inflorescences (3–5 cm) were selected. GV3101 harboring pEGOE35S-AmCS was cultured in LB liquid medium (50 mg/L hygromycin + 50 mg/L rifampicin; 28 °C, 200 rpm) to OD_600_ = 0.8 ± 0.05, centrifuged (4,000 × g, 10 min, 4 °C), and resuspended in induction medium (10 mM MES, 10 mM MgCl_2_, 200 μM AS, pH 5.6) to OD_600_ = 0.8 ± 0.05. After 2 h incubation (28 °C, 200 rpm), inflorescences were immersed for 5 min in suspension (5% sucrose, 0.02% Silwet L-77, 10 mM MgCl_2_, 10 mM MES pH 5.6). Plants were kept in dark (≥80% RH, 48 h) before returning to normal growth. T0 seeds were surface-sterilized [70% ethanol (1 min) → 20% bleach (0.1% NaOCl, 5 min) → 5× sterile water rinses], sown on 1/2 MS + 50 mg/L hygromycin. Resistant seedlings (7–10 d) were transplanted. Genomic DNA was extracted (CTAB method), and transformants were verified by PCR amplification of *HygR* gene (**Supplementary Table S2**). Hygromycin-resistant T2 plants (showing a 3:1 resistant:sensitive) segregation ratio, with χ² test p > 0.05 indicating no significant deviation from the expected ratio for a single T-DNA insertion) were selfed. Putatively homozygous T2 plants (identified by uniform hygromycin resistance in their T3 progeny) were self-pollinated to produce T3 seeds. Homozygous T3 plants were used to establish lines for subsequent experiments.

#### 2.5.3 Validation of the interaction between pGADT7-CS and pGBKT7-PDK

The protein-protein interaction between AmCS and AmPDK was investigated using the Matchmaker™ Gold Yeast Two-Hybrid System (Clontech). The Saccharomyces cerevisiae strain Y2HGold served as the host, with pGBKT7-PDK as the bait plasmid and pGADT7-CS as the prey plasmid. The coding sequences of AmCS and AmPDK were PCR-amplified. Subsequently the vectors pGBKT7-PDK and pGADT7-CS were constructed via homologous recombination (the assembly systems and procedures are detailed in **Supplementary Table S1** and **Supplementary Table S2**). Yeast transformation was performed using the lithium acetate/polyethylene glycol (LiAc/PEG) method. Briefly, overnight cultures of Y2HGold (OD_600_ ≈ 0.6) were washed with 1.1× TE/LiAc solution and then incubated with heat-denatured carrier DNA (10 mg/mL), 1 μg of each pGBKT7-PDK and pGADT7-CS plasmids, and PEG/LiAc transformation buffer. The transformation mixture was incubated at 30 °C for 30 min, heat-shocked at 42 °C for 15 min, and promptly placed on ice for 5 min. Subsequently, the cells were pelleted, resuspended, and spread on SD/-Leu/-Trp (DDO) medium for incubation at 30 °C for 2–3 days. To validate the interactions, single colonies from DDO plates were resuspended in sterile water (OD600 = 0.1) and serially diluted (10–1 to 10-3). Aliquots (10 μL) of each dilution were spotted onto: (i) SD/-Leu/-Trp/X (DDO/X; containing 80 μg/mL X-α-Gal) and (ii) SD/-Ade/-His/-Leu/-Trp/X/A (QDO/X/A; containing 80 μg/mL X-α-Gal and 0.5 μg/mL Aureobasidin A). Four control groups were included: positive control (Y2HGold [pGBKT7-p53 + pGADT7-T]), bait autoactivation control (Y2HGold [pGBKT7-PDK + pGADT7]), prey autoactivation control (Y2HGold [pGBKT7 + pGADT7-CS]), and nonspecific interaction control (Y2HGold [pGBKT7-Lam + pGADT7-CS]). Following incubation at 30 °C for 3–5 days, positive protein-protein interactions were confirmed by the growth of blue colonies on QDO/X/A medium, typically observed at dilutions of 10–^1^ to 10^-3^. Each experiment was conducted with three independent biological replicates.

#### 2.5.4 Comparative physiological and metabolic analysis of *AmCS*-overexpressing lines

A comparative study was conducted to assess the physiological and metabolic characteristics of Wild-type (WT) and homozygous *AmCS*-overexpressing lines. Seed aging experiments were performed following the protocol outlined by Chen et al. (2012) with specific modifications. Initially, 0.2 g of seeds were placed in 4-cm Petri dishes within a desiccator containing saturated KCl solution to maintain 80% relative humidity, and then equilibrated at 20 °C for 3 days in a controlled seed aging chamber. Subsequently, the temperature was raised to 40 °C for an additional 3 days. Aged transgenic seeds were then equilibrated in a desiccator with saturated MgCl_2_ solution (33% RH) for 72 h, followed by surface sterilization using 75% ethanol for 30 seconds and 2% NaClO for 10 min. These treated seeds were then sown on 0.5×MS solid medium with a pH of 5.8 and 0.8% agar, with 50 seeds per 9-cm dish. Three biological replicates were cultured at 22 °C under 16-h light/8-h dark cycles. The germination rates and vigor, defined by radicle emergence of ≥1 mm, were meticulously documented for analysis.

Subsequently, for enzymatic and metabolic profiling, a comprehensive analysis was conducted, which included the assessment of antioxidant enzyme activities (SOD; Kit No. BC5165) and catalase (CAT; Kit No. BC0205) measured through microplate assays, as well as hydrogen peroxide quantification (H2O2; Kit No. BC3595), peroxidase activities (POD; Kit No. BC0090) and ascorbate peroxidase (APX; Kit No. BC0220) determined spectrophotometrically. Furthermore, acetyl-CoA content (Kit No. BC0980), CS, IDH activities, and ATP levels were determined as outlined in Section 2.2. Oxidative stress markers (malondialdehyde, MDA) content were assessed using a plant MDA detection kit (Kit No. A003-1, Nanjing Jiancheng Bioengineering Institute), while cellular respiration rates were measured following the methodology described by Benamar (Benamar et al., 2003). Additionally, the nicotinamide adenine dinucleotide phosphate (NADPH/NADP^+^) ratios were quantified utilizing a commercial assay kit (Kit No. MAK038, Sigma-Aldrich). For NADP/NADPH analysis, frozen samples were homogenized in ice-cold extraction buffer, followed by centrifugation at 12,000 × g for 10 min at 4 °C. The absorbance of the supernatant was then measured at 340 nm, with oxidized NADP^+^ level determined by subtracting NADPH from total NADP content. All biochemical assay kits were obtained from Beijing Solarbio Science & Technology Co., Ltd., unless stated otherwise.

Total RNA was extracted from seeds, roots, and leaves utilizing the FastPure Universal Plant RNA Kit (Vazyme, RC411), followed by cDNA synthesized using HiScript II Reverse Transcriptase (Vazyme, R212). Gene expression levels were quantified by qPCR using the method described by Men et al. (2025). Gene expression fold changes were determined via the 2^-ΔΔCt^ approach (Livak and Schmittgen, 2001), three independent biological replicates, each with three technical replicates. Normality (Shapiro-Wilk) and equal variance ((Levene’s) tests validated ANOVA assumptions prior to analysis, followed by Tukey’s HSD multiple comparisons (α = 0.05) in SPSS 27. Data visualization was performed in Origin 2024.

## 3 Results

### 3.1 Effects of spermidine priming on seed germination in *A. mongolicum*

Our investigation of *A. mongolicum* seeds with varying storage durations (**Supplementary Figure S1A**) revealed a significant time-dependent decline in germination rates (*P* < 0.05). Freshly harvested seeds (0-year storage) displayed a germination rate of 79.00%, which notably declined to 39.00% after 4 years of storage. Application of exogenous Spd significantly enhanced germination rates across all storage periods (*P* < 0.05), resulting in relative increases of 13.08%, 20.45%, and 29.05%, respectively, compared to untreated controls. Notably, seeds with lower initial viability demonstrated a more pronounced response to Spd treatment. Moreover, the promotive effect of Spd on germination is independent of changes in osmotic pressure and pH conditions (**Supplementary Figure S1B**). These results indicate the effective role of Spd in alleviating seed deterioration and significantly enhancing germination potential in aged seeds, with notable restorative effects observed in low-vigor seeds. This study contributes a foundational basis for the development of long-term storage strategies for *A. mongolicum* seeds.

### 3.2 Transcriptome sequencing statistics and quality control analysis

Transcriptome sequencing of *A. mongolicum* seeds stored for varying durations yielded 121.46 Gb of high-quality clean data (≥6 Gb per sample), characterized by exceptional sequencing quality metrics (Q20 ≥97%, Q30 ≥92%, and error rates <1%). The GC content remained consistent (>45%; **Supplementary Table S3**). *De novo* assembly produced 401,059 transcripts and 208,598 unigenes, indicating a high level of assembly completeness (N50 = 1196/1351, N90 = 342/509; **Supplementary Figure S2A**). The C-value (254), an indicator of assembly continuity, surpassed the typical plant transcriptome standards (>200), further validating the assembly integrity (**Supplementary Figure S2B**). Principal component analysis (PCA) revealed that PC1 and PC2 explained 30.06% and 12.82% of the total variance, respectively (**Supplementary Figure S2C**). Notably, PC1 accounted for over 30% of the variance, suggesting its efficacy in capturing significant biological variation. A sample correlation heatmap confirmed robust intra-group reproducibility and distinct inter-group differences (**Supplementary Figure S2D**). Collectively, these results affirm the high reliability of our sequencing data for subsequent biological analyses.

### 3.3 Identification and functional annotation of differentially expressed genes

**Figure 1:**
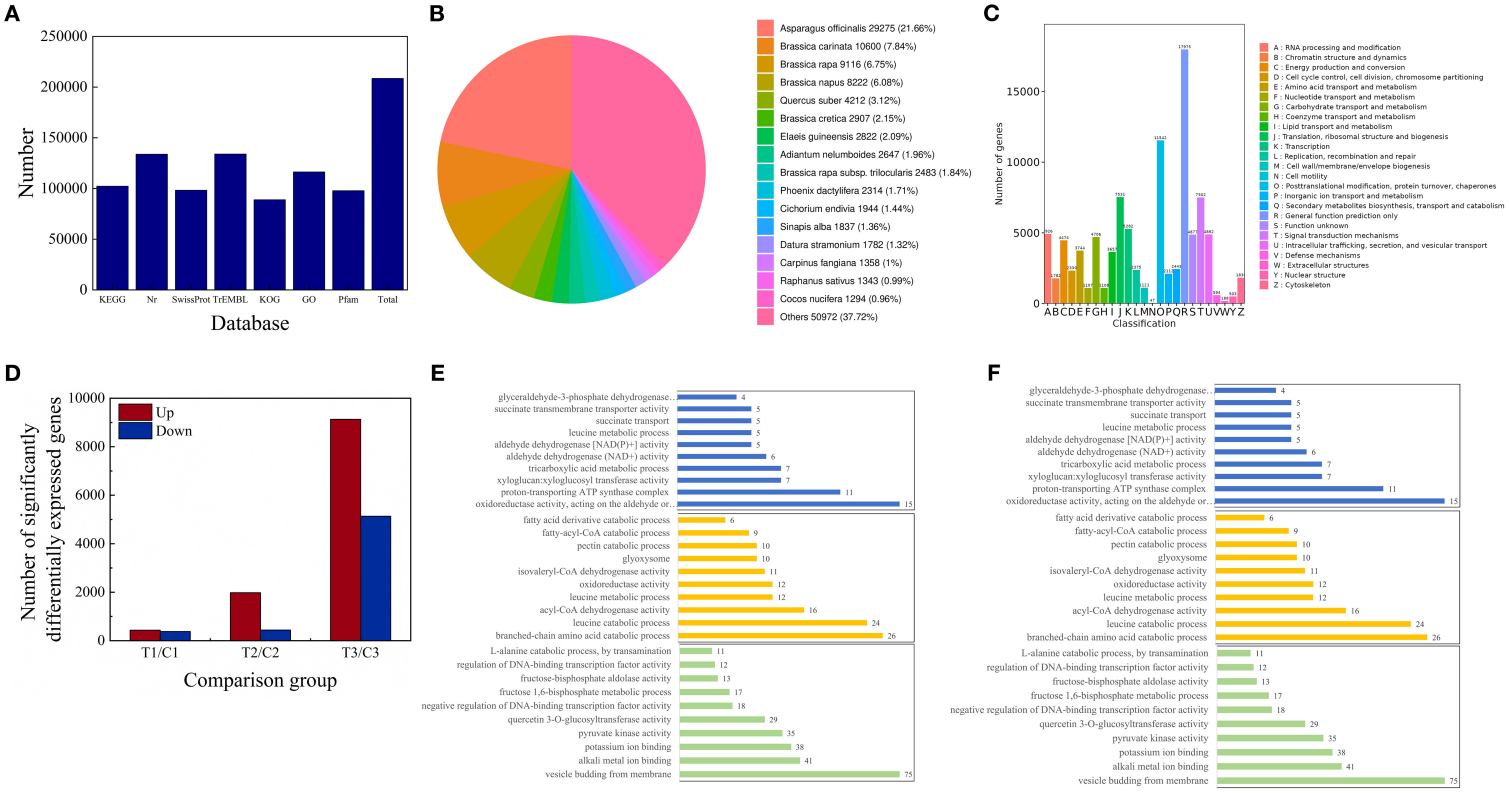
Functional analysis of DEGs in *A. mongolicum* seeds under Spd treatment. **(A)** Functional annotation profile of DEGs. **(B)** Phylogenetic distribution of homologous species (E-value < 1e-5). **(C)** KOG functional classification of DEGs (x-axis: KOG category abbreviations; y-axis: gene counts; color coding represents functional categories with legend). **(D)** Venn diagram analysis of DEGs. **(E)** Enrichment analysis of KEGG pathways (y-axis: pathway names; x-axis: gene counts; color-coded by experimental groups (T1 vs C1: blue; T2 vs C2: orange; T3 vs C3: green). The top 10 significantly enriched pathways are shown for each group). **(F)** Enrichment analysis of GO terms (y-axis: GO terms; x-axis: gene counts; color-coded by comparison groups (T1/C1: blue; T2/C2: orange; T3/C3: green). The top 10 significantly enriched terms are shown for each group).

A comprehensive functional annotation of 208,598 unigenes was performed across seven major databases, yielding substantial annotation coverage (KEGG: 49.06%, NR: 64.16%, Swiss-Prot: 47.11%, TrEMBL: 64.23%, KOG: 42.62%, GO: 55.8%, and Pfam: 46.92%; **Figure 1A**). Comparative analysis of homologous species revealed the highest genomic similarity between *A. mongolicum* and *Asparagus officinalis* (E-value <1e-5; **Figure 1B**), providing evolutionary context for the exploration of seed aging mechanisms. DEGs were identified using stringent criteria (FDR <0.05, |log2FC| ≥2), revealing significant alterations in gene expression profiles induced by Spd treatment, with effects varying based on storage duration (T1/C1: 814; T2/C2: 2,419; T3/C3: 14,263 DEGs; **Figure 1C**). This temporal expression pattern indicates that Spd triggers progressive transcriptome remodeling during seed aging. KOG classification revealed significant enrichment (hypergeometric test, FDR <0.05) of DEGs in post-translational modification, protein turnover, and lipid metabolism (**Figure 1D**). Subsequent KEGG pathway analysis unveiled the upregulation of energy metabolism pathways (such as glycolysis, TCA cycle, and oxidative phosphorylation; **Figure 1E**), suggesting that Spd enhances mitochondrial energy production. Furthermore, pathway enrichment in phenylpropanoid and flavonoid biosynthesis implies antioxidant capacity reinforcement. GO enrichment analysis (FDR <0.05) highlighted that DEGs were predominantly associated with: Molecular functions (including hydrolase activity, oxidoreductase activity, and ion binding), Biological processes (such as branched-chain amino acid catabolism, TCA cycle, and fatty acid β-oxidation), and Cellular components (encompassing mitochondria, peroxisomes, and proton-transporting ATP synthase complex) (**Figure 1F**). These results support a multi-pathway mechanism wherein Spd retards seed aging by orchestrating energy metabolism reprogramming, maintaining proteostasis, regulating lipid metabolism, and activating oxidative stress defenses. This study provides molecular-level insights into Spd-mediated seed longevity extension in xerophytic plants.

### 3.4 Quality control and differential metabolite screening in energy metabolomics

**Figure 2:**
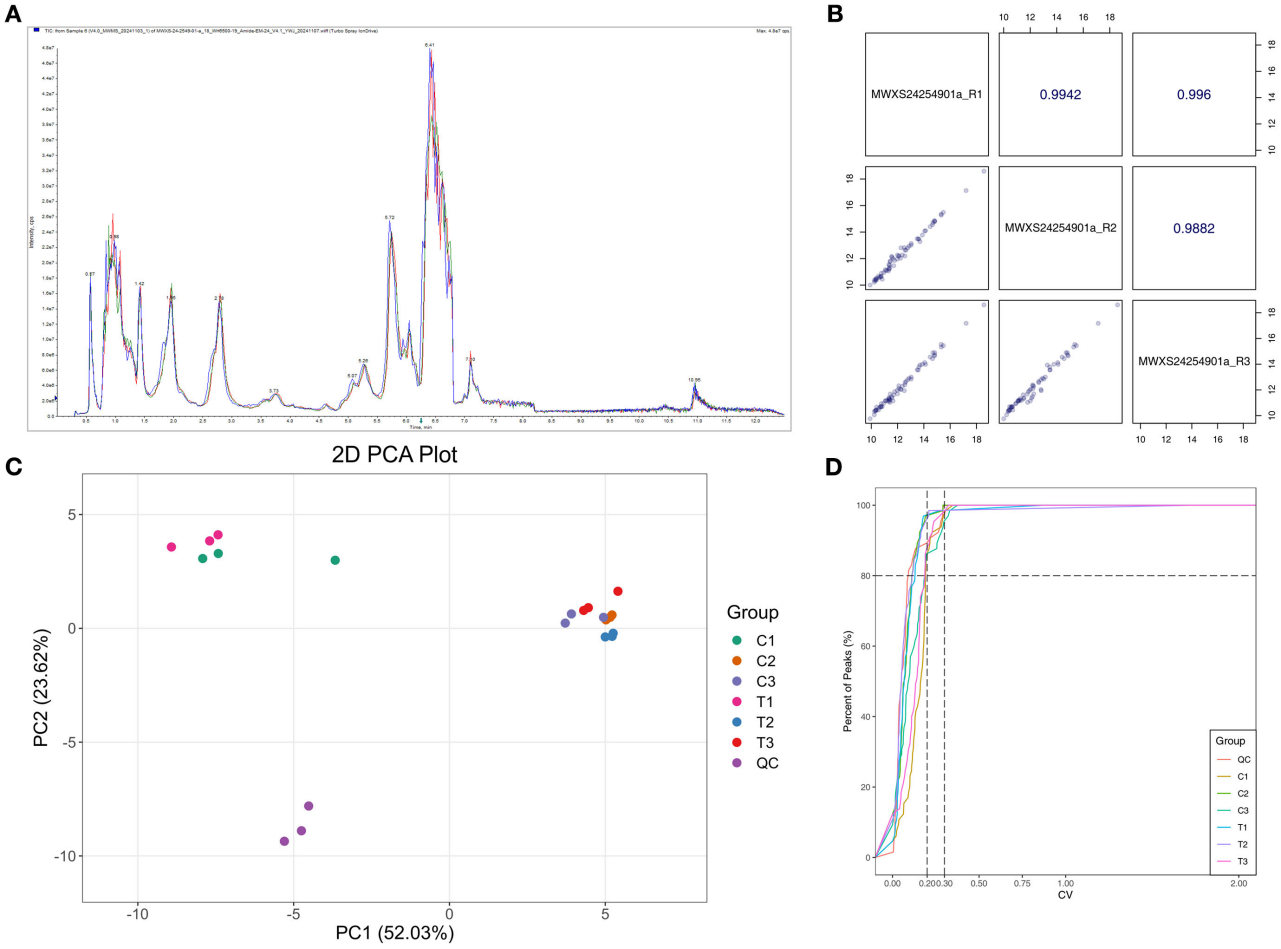
Quality control analysis of targeted energy metabolomics data. **(A)** Total ion chromatograms (TIC). **(B)** Correlation analysis of quality control (QC) samples: diagonal panels indicate QC sample names; lower-left panels display metabolite abundance scatter plots (points represent metabolites); upper-right panels show Pearson correlation coefficients (r-values). **(C)** Principal component analysis (PCA) score plot: PC1 and PC2 (first/second principal components) with variance percentages; sample points are color-coded by group. **(D)** Metabolite coefficient of variation (CV) cumulative distribution: x-axis: CV thresholds; y-axis: proportion below thresholds; vertical dashed lines at CV = 0.2/0.3; horizontal dashed line at 80% cutoff.

This study employed a targeted energy metabolomics approach with rigorous quality control measures to ensure data reliability. The total ion chromatograms (TIC) exhibited consistent retention times and peak overlap across all samples (**Figure 2A**). Both Pearson correlation analysis (|r| > 0.99; **Figure 2B**) and principal component analysis (PC1 = 52.03%, PC2 = 23.62%; **Figure 2C**) confirmed excellent experimental reproducibility without notable batch effects. Quality control (QC) samples demonstrated that 83.1% of metabolites had coefficient of variation (CV) values below 30% (**Figure 2D**), indicating high data stability. By applying stringent criteria (VIP > 1, |log_2_FC| ≥ 1.5 or ≤ 0.67, *P* < 0.01), 24 significantly altered metabolites were identified among the 65 detected compounds. These differential metabolites were categorized into four major classes: nucleotides (e.g., ATP), phosphosugars (e.g., glucose-6-phosphate), organic acids and derivatives (e.g., citrate, malate), and carbohydrates (e.g., α-D-glucose) (**Supplementary Table S4**). Notably, the significant alterations (*P* < 0.05) observed in crucial glycolytic intermediates such as citrate, malate, and acetyl-CoA indicate a substantial metabolic reorganization affecting both the TCA cycle and glycolysis pathway, potentially playing a pivotal role in the regulation of cellular energy homeostasis.

### 3.5 Dynamic remodeling of carbohydrate catabolic pathways during natural aging of *A. mongolicum* seeds

**Figure 3:**
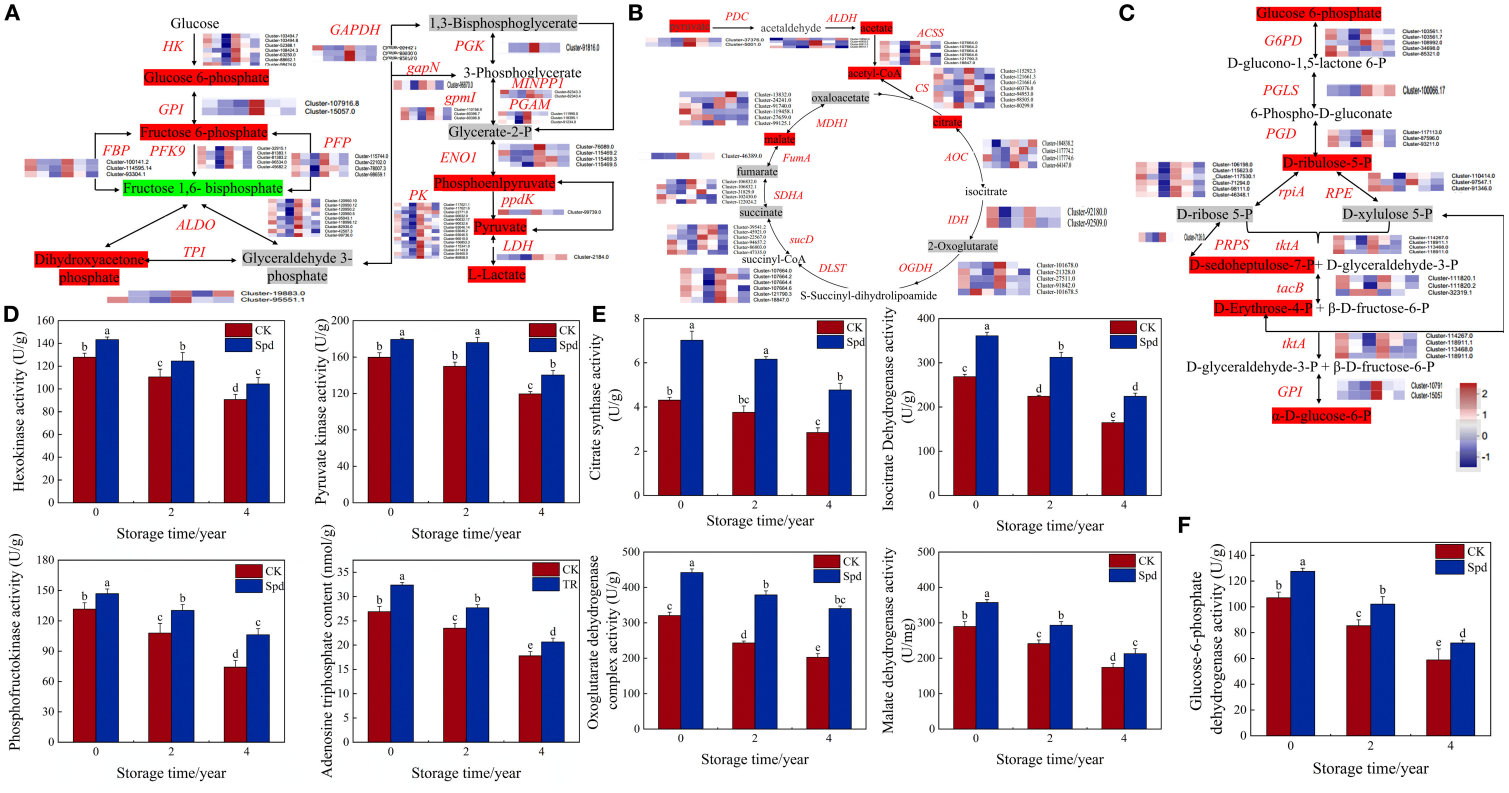
Regulatory effects of Spd treatment on carbohydrate metabolic pathways in *A. mongolicum* seeds. **(A)** Schematic representation of the glycolytic pathway. **(B)** Schematic overview of the TCA cycle. **(C)** Diagram of the pentose phosphate pathway. **(D)** Activity changes of key EMP enzymes (U/g) during different storage periods. **(E)** Activity changes of core TCA cycle enzymes (U/g) across storage durations; **(F)** Activity variations of key PPP enzyme (U/g) under different storage conditions. Data are presented as means ± SD (n = 3 biological replicates). Gene expression levels are quantified by FPKM, with sample ordered as: C1, C2, C3 (control) and T1, T2, T3 (Spd-treated). Different lowercase letters indicate statistically significant differences (P < 0.05, one-way ANOVA with Tukey’s test).

This study employed an integrated multi-omics approach to systematically investigate the regulatory effects of Spd on sugar metabolism in *A. mongolicum* seeds. Through a combination of enzymatic assays, targeted metabolomics, and transcriptome sequencing, we unveiled dynamic reprogramming of glycolysis (EMP), the TCA cycle, and the pentose phosphate pathway (PPP) during seed aging. Transcriptomic analysis identified 16 classes of DEGs (**Figure 3A**; **Supplementary Table S5**), including key enzymes such as 7 hexokinases (*HK*), 5 6-phosphofructokinases (*PFK*), 15 pyruvate kinases (*PK*), 9 fructose-1,6-bisphosphate aldolases (*ALDO*), 4 enolases (*ENO1*), 2 glucose-6-phosphate isomerases (*GPI*), 3 fructose-1,6-bisphosphatases (*FBP*), 4 pyrophosphate-dependent phosphofructokinases (*PFP*), 2 triosephosphate isomerases (*TPI*), 1 phosphoglycerate kinase (*PGK*), 1 glyceraldehyde-3-phosphate dehydrogenase (*gapN*), 2 inositol polyphosphate phosphatases (*MINPP1*), 3 2,3-bisphosphoglycerate-independent phosphoglycerate mutases (*gpmI*), 3 2,3-bisphosphoglycerate-dependent phosphoglycerate mutases (*PGAM*), 1 pyruvate phosphate dikinase (*ppdK*), and 1 L-lactate dehydrogenase (*LDH*). Spd treatment significantly upregulated these genes, as confirmed by enzymatic assays showing marked increases in HK, PFK, and PK activities (P < 0.05) (**Figure 3D**; **Supplementary Table S5**). Metabolomic analysis revealed that Spd upregulated six glycolytic intermediates (α-D-glucose-6-phosphate, β-D-glucose-6-phosphate, dihydroxyacetone phosphate, phosphoenolpyruvate, pyruvate, and L-lactate) while downregulating fructose 1,6-bisphosphate. Importantly, Spd reversed age-related ATP depletion (**Figure 3A**), indicating enhanced cellular energy supply through glycolytic activation.

We further characterized Spd-mediated regulation of the TCA cycle (**Figure 3B**; **Supplementary Table S5**), identifying 11 gene classes, including components of the pyruvate dehydrogenase complex (2 E1 components (*aceE*) and 6 E2 components (*DLAT*), core TCA enzymes: 7 citrate synthase (*CS*), 4 aconitate hydratase (*AOC*), 2 isocitrate dehydrogenase (*IDH*), 5 2-oxoglutarate dehydrogenase E1 components (OGDH), and 6 dihydrolipoamide succinyltransferase (E2 components, DLST), as well as downstream enzymes: 6 succinyl-CoA synthetase (*αsucD*), 5 succinate dehydrogenase flavoprotein (*SDHA*), 1 fumarate hydratase (*fumA*), and 6 malate dehydrogenase (MDH1). FPKM quantification revealed that natural aging led to downregulation of these genes, while Spd treatment restored their expression levels (*P* < 0.05). Enzymatic activities were consistent with the gene expression profiles (**Figure 3E**). Metabolomic analysis confirmed Spd-enhanced accumulation of TCA intermediates (acetate, acetyl-CoA, citrate, malate, and pyruvate; *P* < 0.05), with other metabolites remaining stable (*P* > 0.05), highlighting targeted activation of mitochondrial energy metabolism.

Furthermore, we elucidated Spd’s novel role in delaying seed aging through PPP activation (**Figure 3C**; **Supplementary Table S5**), identifying 10 gene classes, including 5 glucose-6-phosphate dehydrogenase (*G6PD*), 1 6-phosphogluconolactonase (*PGLS*), 3 6-phosphogluconate dehydrogenase (*PGD*), 6 ribose-5-phosphate isomerase A (*rpiA*), 3 ribulose-phosphate 3-epimerase (*RPE*), 1 ribose-phosphate pyrophosphokinase (*PRPS*), 4 transketolase (*tktA*), 3 transaldolase (*talB*), and 2 glucose-6-phosphate isomerase (*GPI*). The expression pattern of the rate-limiting enzyme G6PD correlated with its enzymatic activity (**Figure 3F**). Metabolomic analysis revealed increased levels of key PPP intermediates (glucose 6-phosphate, ribulose 5-phosphate, D-sedoheptulose 7-phosphate, D-erythrose 4-phosphate, and α-D-glucose 6-phosphate; *P* < 0.05). These findings collectively establish Spd’s tripartite anti-aging mechanism: (i) enhancing glycolytic flux via HK-PFK-PK cascade, (ii) maintaining TCA cycle homeostasis, and (iii) upregulating G6PD to boost PPP flux for NADPH and nucleotide precursor supply. Notably, Spd activates the oxidative PPP phase through the G6PD-PGD axis while inducing *tktA* and *talB* expression to direct carbon skeletons towards nucleotide synthesis in the non-oxidative phase. This novel mechanism concurrently increases NADPH (antioxidant) and nucleotide precursor (DNA repair) supplies, offering fresh insights into the metabolic regulation of seed aging.

### 3.6 Cloning and functional characterization of the *AmCS* gene

**Figure 4:**
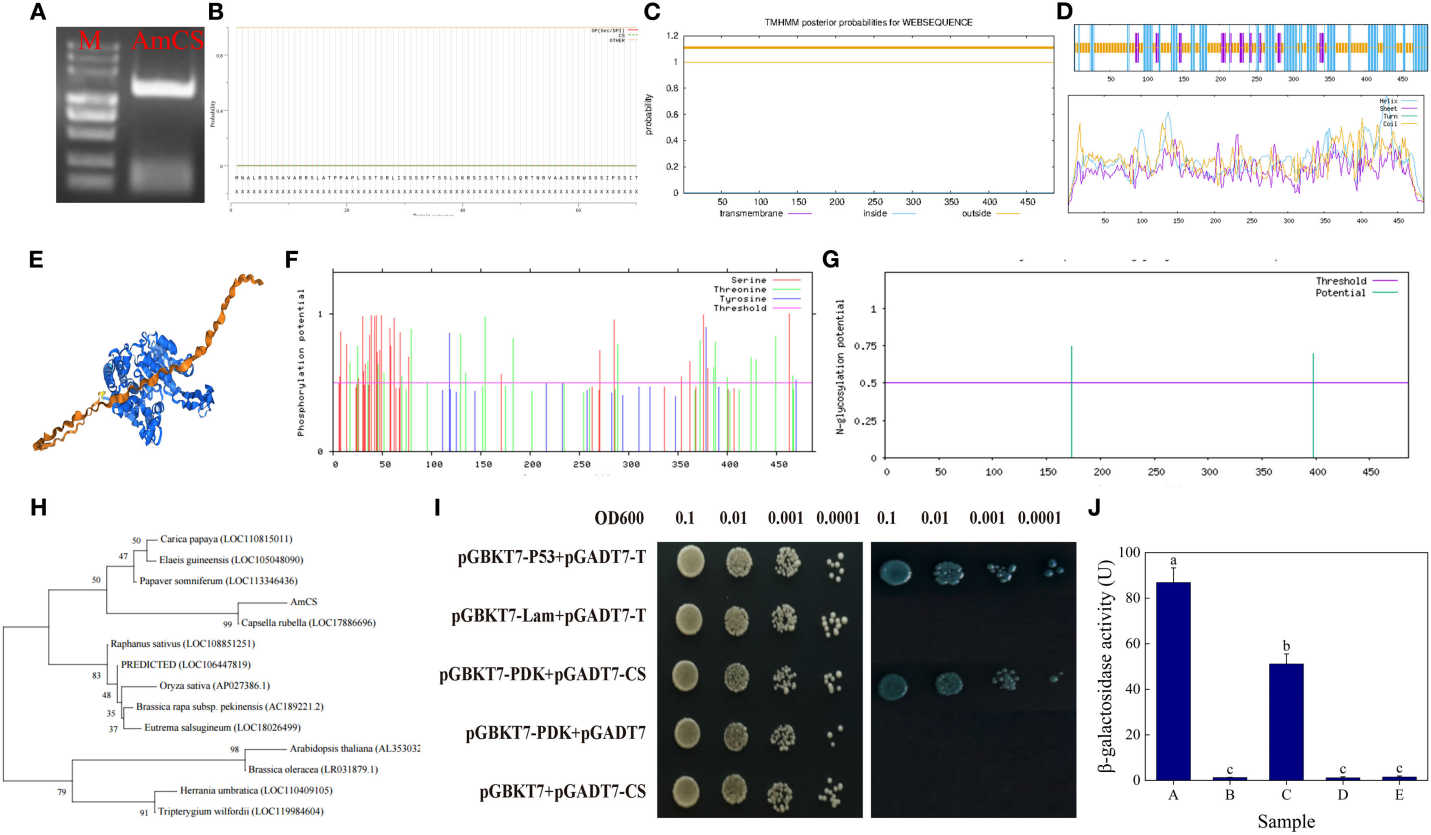
Molecular characterization and interaction analysis of AmCS. **(A)** PCR amplification of the AmCS gene. M: 5,000 bp DNA marker; AmCS: 1,461 bp product. **(B)** Signal peptide prediction of AmCS. **(C)** Transmembrane domain prediction of AmCS. **(D)** Secondary structure prediction of AmCS. **(E)** Homology modeling of the AmCS tertiary structure. **(F)** Phylogenetic analysis of AmCS. **(G)** Phosphorylation site prediction of AmCS. **(H)** N-glycosylation site prediction of AmCS. **(I)** Protein-protein interaction between AmCS and AmPDK. **(J)** β-Galactosidase Activity. A: Positive control; B: Negative control; C: Experimental group; D and F: Empty vector control.

We cloned and characterized a citrate synthase (CS) gene, named *AmCS*, from *A. mongolicum* seeds (**Figure 4A**), comprising a 1,461-bp open reading frame (ORF) encoding a 487-amino acid protein (127.1 kDa, pI 8.7). Bioinformatics analysis revealed the absence of signal peptides or transmembrane domains in AmCS (**Figures 4B, C**) and indicated a predominance of α-helical structures in its composition (**Figures 4D, E**). Phylogenetically, AmCS exhibits the highest homology with CS from *Corylus avellana*, *Pistacia vera*, and *Citrus* species (**Figure 4F**). Post-translational modification (PTM) prediction identified 43 phosphorylation and 2 N-glycosylation sites (**Figures 4G, H**), suggesting potential PTM-mediated regulatory mechanisms. Yeast two-hybrid (Y2H) assays demonstrated a specific interaction between AmCS and pyruvate dehydrogenase kinase (AmPDK) (**Figure 4I**; **Supplementary Figure S3**). Quantitative yeast two-hybrid assays based on β-galactosidase activity (ONPG hydrolysis) further supported a specific interaction between AmCS and AmPDK, consistent with the qualitative results (**Figure 4J**). Integrated KEGG analysis suggests that the AmCS-AmPDK module coordinates TCA cycle flux (increasing ATP production) and pyruvate dehydrogenase complex (PDC) activity (reducing ROS production) to maintain metabolic homeostasis.

### 3.7 Generation and functional validation of *AmCS*-overexpressing lines

**Figure 5:**
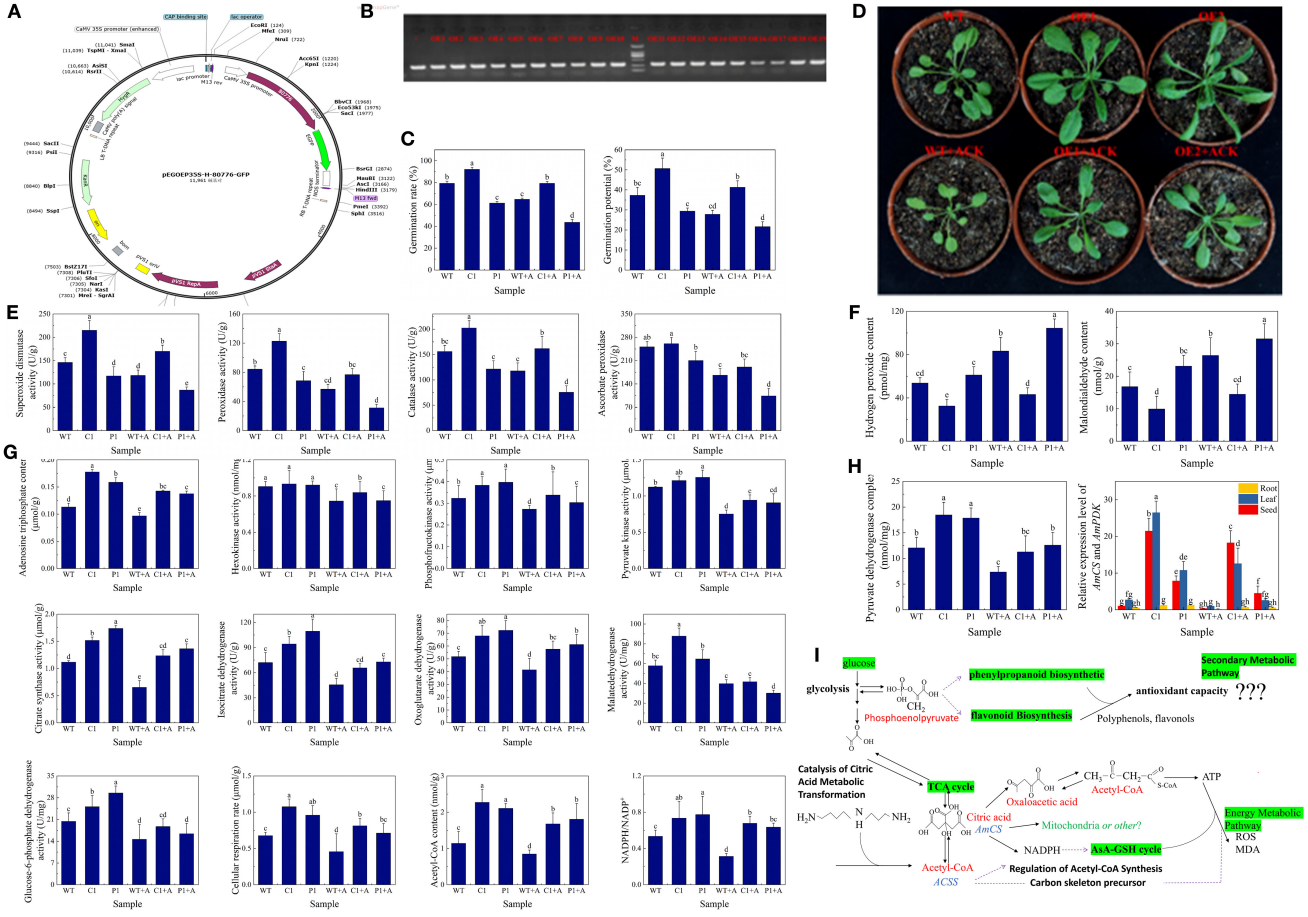
Molecular characterization and anti-aging functional analysis of AmCS-overexpressing transgenic lines. **(A)** Illustration of the pEGOE35S-AmCS overexpression vector structure (LB/RB: T-DNA borders; 35S: CaMV 35S promoter; 80776: AmCS gene; HygR: hygromycin resistance gene). **(B)** PCR identification of transgenic lines (+: plasmid positive control; –: H2O negative control; OE1–OE19: overexpression lines; 385 bp hygromycin gene fragment). **(C)** Germination rate and germination potential of AmCSoverexpressing Arabidopsis seeds. ACK: artificially aged Arabidopsis thaliana seeds. **(D)** Phenotypic analysis of seedling growth. **(E)** Antioxidant enzyme activities (U·g^-1^ protein). **(F)** H2O2 and MDA contents (H2O2: pmol/mg; MDA: nmol/g); **(G)** Pyruvate dehydrogenase complex (PDC) activity and AmCS expression (RTqPCR). **(H)** Enzyme activities and metabolite levels in carbohydrate catabolic pathways. **(I)** Model for AmCS-mediated anti-aging mechanism. Data presented as means ± SD (n = 3 biological replicates). Different lowercase letters indicate statistically significant differences (evaluated using one-way ANOVA, *P* < 0.05).

This study systematically elucidated the pivotal role of citrate synthase in seed germination and aging resistance through the generation of *Arabidopsis* lines overexpressing AmCS (**Figure 5A**). Molecular characterization identified 19 stable overexpression lines (**Figure 5B**). The germination rates of the OE1 and OE2 lines increased significantly by 16.00% and 15.14%, respectively, compared to the WT (**Figure 5C**), indicating that AmCS overexpression enhances seed germination. Phenotypic assessments revealed accelerated germination and more vigorous seedlings growth in the overexpression lines (**Figure 5D**). Under oxidative stress conditions, the OE1 lines exhibited 46.06%, 45.60%, and 29.60% increases in SOD, POD, and CAT activities, respectively (**Figure 5E**), accompanied by significant reductions in H_2_O_2_ (35.2%) and MDA (40.40%) contents (**Figure 5F**), confirming an augmented antioxidant capacity. Tissue-specific expression analysis revealed the highest AmCS transcript levels in leaves (13.85-fold and 1.41-fold higher compared to roots and seeds, respectively), while artificial aging treatments significantly suppressed its expression (**Figure 5G**). Metabolic profiling revealed significant increases in acetyl-CoA levels (91.81%), CS activity (45.77%), ATP production (46.37%), cellular respiration rates (50.26%), and NADPH/NADP^+^ (1.41-fold) ratios in the overexpression lines (**Figure 5H**). Collectively, these results indicate that AmCS regulates seed vigor through a dual mechanism: (i) enhancing energy production via the TCA cycle and (ii) maintaining redox homeostasis to delay aging (**Figure 5I**). This study identifies AmCS as a potential genetic target for enhancing seed longevity in agricultural crops.

## 4 Discussion

### 4.1 Effects of exogenous Spd treatment on germination of aged *A. mongolicum* seeds

Seed vigor preservation is a crucial factor in crop production (Pirredda et al., 2024). Our study demonstrates that prolonged storage significantly inhibits the germination capacity of *A. mongolicum* seeds (**Supplementary Figure S1**), consistent with findings in aged seeds of Poaceae (Zhang et al., 2023) and Fabaceae (da Silva et al., 2023) species. Increasing evidence underscores the essential role of polyamines, particularly Spd, as endogenous anti-aging signaling molecules in seed vigor regulation (Huang et al., 2018; Hu et al., 2020; Ma, 2023). Notably, exogenous Spd application enhanced germination rates by 13% to 29% in aged *A. mongolicum* seeds (**Supplementary Figure S1**), demonstrating significantly stronger restorative effects compared to aged sorghum and rice seeds (Zhang et al., 2023; Hu et al., 2020). This disparity suggests potential species-specific characteristics in either polyamine signaling sensitivity or germination regulatory networks of *A. mongolicum*. While our findings support the germination-enhancing effects of Spd as previously observed in sorghum (Zhang et al., 2023) and rice (Hu et al., 2020), the underlying mechanisms require further investigation. This study provides substantive evidence that exogenous Spd treatment effectively mitigates aging-induced germination impairment, laying the groundwork for the development of polyamine-based technologies for seed anti-aging strategies. These findings position Spd as a promising biochemical agent for preserving seed vigor in xerophytic species.

### 4.2 Multi-omics analysis reveals exogenous Spd-mediated seed vigor improvement in *A. mongolicum*

Integrated multi-omics approaches have emerged as a robust framework for elucidating plant stress responses (Zagorščak et al., 2025). Through coordinated transcriptomics (RNA-seq), targeted metabolomics (LC-MS/MS), and enzymatic assays, we systematically elucidated how Spd restores vigor in aged *A. mongolicum* seeds via dual mechanisms: reprogramming of energy metabolism and alleviation of oxidative stress (**Supplementary Figure S1**). In addition to confirming Spd’s role in activating carbohydrate metabolism, our multi-omics data unveiled a cascade of responses, spanning from transcriptional regulation to metabolic network remodeling, as evidenced by KEGG enrichment (**Figure 1**) and metabolic flux analysis (**Figure 2**; **Supplementary Table S4**). Energy dysregulation stands out as a characteristic feature of seed aging. Prolonged storage significantly reduced activities of key enzymes in EMP, the TCA cycle, and the PPP—including PFK, GAPDH, PK, DLD, IDH, and MDH (*P* < 0.05, **Figure 2**), consistent with findings in oat (Li et al., 2022) and elm (Li et al., 2024). Transcriptomics confirmed the downregulation of corresponding genes and impaired ATP production (*P* < 0.05; **Figure 3**, **Supplementary Table S5**). Serving as a precursor for coenzyme A synthesis via pantothenate (Webb and Smith, 2011; Davaapil et al., 2014), Spd upregulated *ACSS* expression (**Figure 3**, **Supplementary Table S5**), expanding the acetyl-CoA pool. Concurrently, it enhanced glycolysis through rate-limiting enzymes (HK, PFK, and PK), accelerating pyruvate production and elevating TCA intermediates (citrate and α-ketoglutarate), ultimately augmenting ATP levels (*P* < 0.05). These observations are in line with previous reports in sorghum (Zhang et al., 2023) and *Arabidopsis* (Takahashi et al., 2019), suggesting an evolutionarily conserved role of polyamine-mediated metabolic regulation. LC-MS/MS-based metabolomics revealed Spd-induced phosphoenolpyruvate (PEP) accumulation (*P* < 0.05), which was channeled into the phenylalanine pathway, resulting in elevated flavonoid levels. Transcriptomics confirmed enrichment of DEGs in phenylpropanoid and flavonoid biosynthesis (FDR < 0.05), with upregulated *CYP75A*, HPPR, *DFR*, and *C12RT1* expression correlating with metabolite changes (**Supplementary Table S5**). Given the direct ROS-scavenging capability of endogenous polyamines like Spd, our data demonstrated a significant reduction in H_2_O_2_ and MDA levels (*P* < 0.05), attributable to their amino groups neutralizing hydroxyl radicals and superoxide anions. This multi-faceted regulatory mechanism establishes a “metabolic substrate redistribution–antioxidant defense–energy supply” triad, offering a novel framework for comprehending the spatiotemporal dynamics of polyamine-mediated seed vigor regulation.

### 4.3 Functional characterization of *AmCS* in plant stress responses

Citrate synthase, as the key rate-limiting enzyme of the TCA cycle, mediates the condensation of acetyl-CoA and oxaloacetate to generate citrate, thus playing a central role in plant energy metabolism, carbon-nitrogen balance, and stress responses (Eprintsev et al., 2018). Apart from its fundamental metabolic function, CS is involved in modulating nitrogen assimilation (Sweetlove et al., 2010), antioxidant defense (Igamberdiev and Eprintsev, 2016), and metal ion detoxification (Anoop et al., 2003) by regulating citrate levels. As the initial committed enzyme of the TCA cycle, CS activity directly determines mitochondrial respiratory efficiency. Our electron microscopy results (**Supplementary Figure S4**) have directly confirmed that this functional decline is closely associated with mitochondrial structural damage, including blurred mitochondrial membranes and the disappearance of inner membrane cristae. In Arabidopsis, CS mutants exhibit growth retardation and mitochondrial dysfunction (Pracharoenwattana et al., 2005), highlighting its critical role in maintaining energy balance. Our study demonstrates that in *AmCS*-overexpressing lines, there was a significant enhancement in TCA cycle flux, evident through increased activities of CS, IDH, and MDH, as well as increased ATP production rates (*P* < 0.05). These findings are consistent with previous reports of CS-mediated respiratory maintenance under salt stress (Zhao et al., 2021), confirming its role in alleviating energy crises. Notably, CS influences redox homeostasis independently of energy metabolism. *AmCS* overexpression significantly raised the NADPH/NADP^+^ ratio (*P* < 0.05), activating the ascorbate-glutathione (ASA-GSH) cycle, while differentially enhancing SOD and CAT activities, thereby reducing H_2_O_2_ and MDA accumulation (**Figures 5E, F, H**, **Supplementary Table S6**). This mechanism corroborates the mitochondrial-cytosolic redox crosstalk model proposed by Igamberdiev and Eprintsev (2016). Our results reveal a dual regulatory mechanism of *AmCS* in seed vigor maintenance: (1) promoting acetyl-CoA entry into the TCA cycle to enhance ATP production, and (2) sustaining antioxidant defense via NADPH regeneration (**Figure 5I**). These observations are consistent with recent studies on light-regulated CS functions (Xu et al., 2023) and validate the oxidative stress sensitivity of cytosolic CS-deficient *Arabidopsis* (Chepelev et al., 2009). Yeast two-hybrid assays confirmed a physical interaction between AmCS and AmPDK (**Figure 4I**). Additionally, KEGG pathway analysis suggests their coordinated regulation of key nodes in pyruvate metabolism, potentially impacting the acetyl-CoA provision. This discovery provides new insights into the interplay between the TCA cycle and glycolysis.

## 5 Conclusions

This study establishes a Spd-AmCS-mediated “energy–redox” coordination network that maintains seed vigor in A. mongolicum. As the central node of this regulatory network, AmCS delays seed aging through dual mechanisms (**Figure 5I**): (i) promoting TCA cycle flux to enhance ATP synthesis efficiency in energy metabolism, and (ii) regulating the NADPH/NADP^+^ ratio to activate the ASA-GSH cycle system for redox homeostasis. Notably, our findings reveal that Spd influences the coupling between the TCA cycle and glycolysis through the interaction of AmCS with AmPDK, providing novel insights into the dynamic balance between energy and oxidative metabolism during seed aging. Future research should prioritize: (i) identifying key amino acid residues in the AmCS-AmPDK interaction using site-directed mutagenesis, (ii) elucidating spatiotemporal patterns of the Spd-AmCS regulatory network through stable isotope-assisted metabolic flux analysis, and (iii) developing AmCS-based gene editing strategies for molecular breeding aimed at preserving seed vigor.

## Data Availability

The datasets presented in this study can be found in online repositories. The names of the repository/repositories and accession number(s) can be found in the article/**Supplementary Material**.

## Supplementary material

SUPPLEMENTARY FIGURE S1 Spermidine priming enhances seed germination in *A. mongolicum* **(A)** Germination rate of *A. mongolicum* seeds under different storage years. **(B)** Spd vs. isoosmotic/pH control: germination comparison. Data are presented as means ± SD (n = 3). Different lowercase letters indicate significant differences (P < 0.05, one-way ANOVA with Tukey’s test). CK: distilled water control; TR: 0. 8 mM spermidine priming treatment.

SUPPLEMENTARY FIGURE S2 Quality assessment of transcriptome assembly and sequencing data **(A)** Transcript and unigene assembly statistics. (B) Assembly completeness assessed by C-value. **(C)** PCA of sample distribution. **(D)** Sample correlation heatmap based on Pearson coefficients (|r|), with color gradient from white (|r|=0) to dark red (|r|=1). Diagonal blocks indicate intra-group reproducibility (|r|>0.95).

SUPPLEMENTARY FIGURE S3 Schematic of the vector construct for interacting proteins. **(A, B)** are schematics of the pGADT7-CS and pGBKT7-PDK vector constructs, respectively.

SUPPLEMENTARY FIGURE S4 Mitochondrial structure in *A. mongolicum* seeds with different storage years observed by TEM. C1: Mitochondrial structure of Allium mongolicum seeds with 0-year storage. C3: Mitochondrial structure of Allium mongolicum seeds with 4-year storage.
