# Supplementary figures and images for: Spermidine improves seed viability in Allium mongolicum by regulating AmCS-mediated metabolic and antioxidant networks
Source: Front Plant Sci. 2025 Oct 8;16:1683362. doi: 10.3389/fpls.2025.1683362 (PMC12540469; doi:10.3389/fpls.2025.1683362)

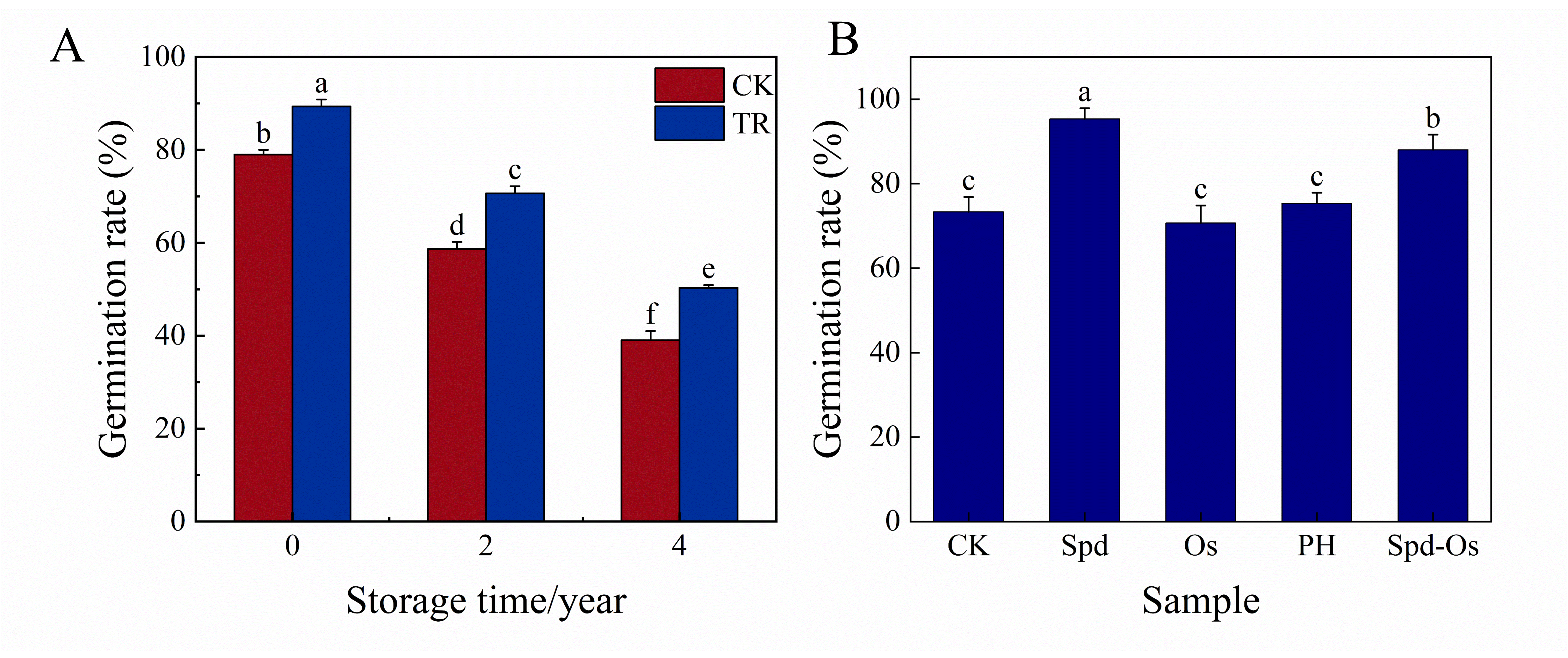

Supplement: SUPPLEMENTARY FIGURE S1 — Spermidine priming enhances seed germination in A. mongolicum (A) Germination rate of A. mongolicum seeds under different storage years. (B) Spd vs. isoosmotic/pH control: germination comparison. Data are presented as means ± SD (n = 3). Different lowercase letters indicate significant differences (P < 0.05, one-way ANOVA with Tukey’s test). CK: distilled water control; TR: 0. 8 mM spermidine priming treatment. [file Image1.tif]

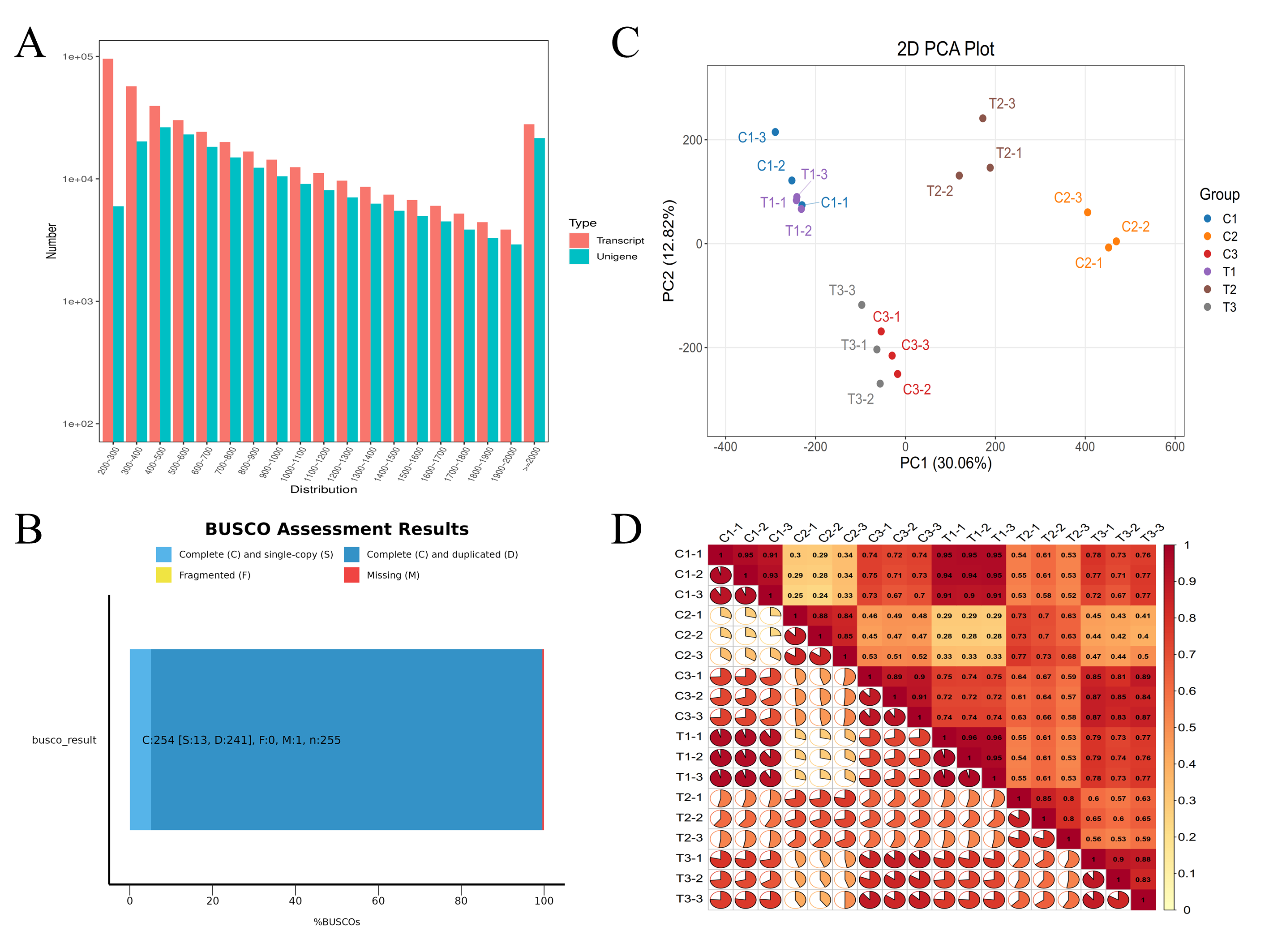

Supplement: SUPPLEMENTARY FIGURE S2 — Quality assessment of transcriptome assembly and sequencing data (A) Transcript and unigene assembly statistics. (B) Assembly completeness assessed by C-value. (C) PCA of sample distribution. (D) Sample correlation heatmap based on Pearson coefficients (|r|), with color gradient from white (|r|=0) to dark red (|r|=1). Diagonal blocks indicate intra-group reproducibility (|r|>0.95). [file Image2.tif]

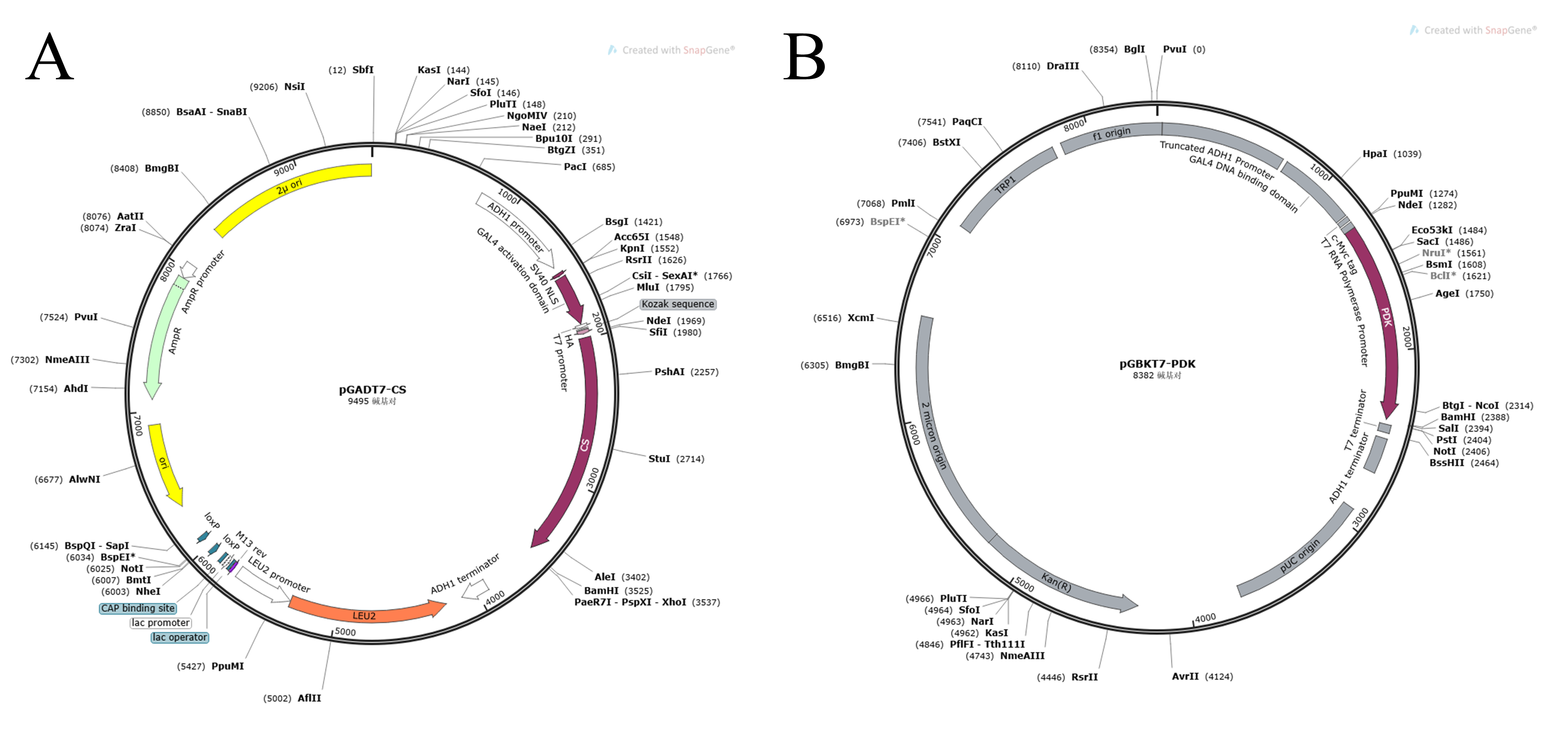

Supplement: SUPPLEMENTARY FIGURE S3 — Schematic of the vector construct for interacting proteins. (A, B) are schematics of the pGADT7-CS and pGBKT7-PDK vector constructs, respectively. [file Image3.tif]

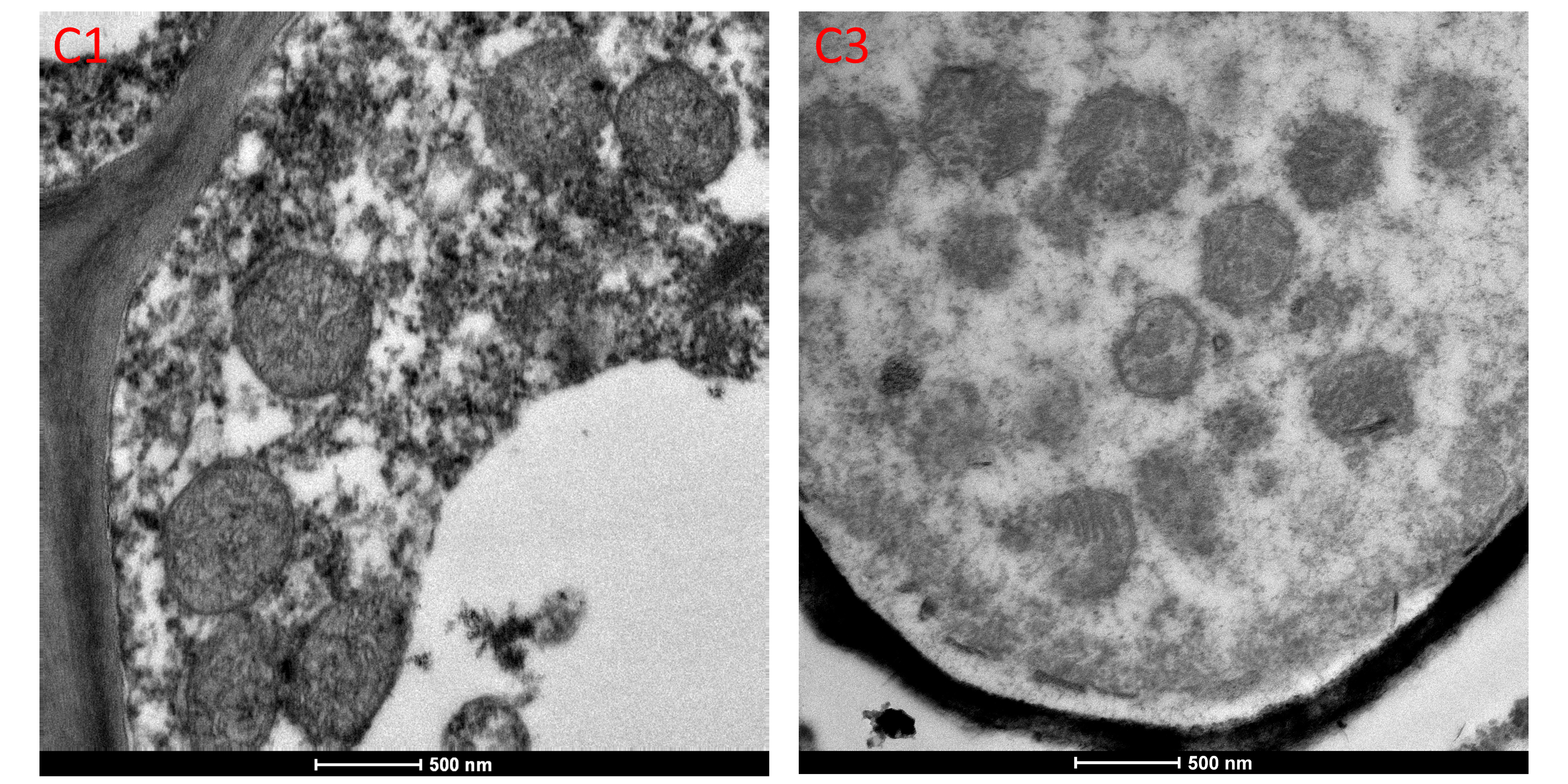

Supplement: SUPPLEMENTARY FIGURE S4 — Mitochondrial structure in A. mongolicum seeds with different storage years observed by TEM. C1: Mitochondrial structure of Allium mongolicum seeds with 0-year storage. C3: Mitochondrial structure of Allium mongolicum seeds with 4-year storage. [file Image4.tif]
