# Supplementary material for: Spermidine improves seed viability in Allium mongolicum by regulating AmCS-mediated metabolic and antioxidant networks
Source: Front Plant Sci. 2025 Oct 8;16:1683362. doi: 10.3389/fpls.2025.1683362 (PMC12540469; doi:10.3389/fpls.2025.1683362)
Supplement: Supplementary file 5 [file Table1.docx]

| Table S1. Primer sequences used in this study | | |
| --- | --- | --- |
| **Name** | **Sequence** | **Purposes** |
| *AmCS*-F | Actag ggtctcG CACC ATGAACGCTCTCCGGTCCAGTTCCGCAGT | Assembly of overexpression plasmids, confirmation of selectable markers, and subcellular localization |
| *AmCS*-F | Actag ggtctcT CGCC CAGGCGAGCCTTGAGGTTGGCCTTCAGCC |  |
| 35S-F | CACGGGGGACTCTTGCCACC |  |
| eGFP-cx | GACACGCTGAACTTGTGG |  |
| HygR-F | TGTAGTGTATTGACCGATTCCTTGC |  |
| HygR-R | GTTCGACAGTGTCTCCGACCTGAT |  |
| *AmCS-*F | ACGGTGTTGGTGTCAAGTGT | Analysis of *AmCS* gene expression |
| *AmCS-*R | GGGGAATGACAATGGGCTCA |  |
| *ACTIN2-*F | 5'-GGCGATGAAGCTCAATCCAAACG-3' | Reference gene |
| *ACTIN2-*R | 5'-GGTCACGACCAGCAAGATCAAGACG-3' |  |
