# Supplementary material for: Spermidine improves seed viability in Allium mongolicum by regulating AmCS-mediated metabolic and antioxidant networks
Source: Front Plant Sci. 2025 Oct 8;16:1683362. doi: 10.3389/fpls.2025.1683362 (PMC12540469; doi:10.3389/fpls.2025.1683362)
Supplement: Supplementary file 6 [file Table2.docx]

Table S2-1. PCR amplification and purification of the target cDNA fragment

| **Components** | **Volume (50 μL)** |
| --- | --- |
| 2× PCR buffer | 25 µL |
| 2mM dNTPs | 10 µL |
| AmCS-F (10 μM) | 1 µL |
| AmCS-R (10 μM) | 1 µL |
| KOD-FX DNA Polymerase (1 U/μL) | 1 µL |
| cDNA (100 ng/μL) | 1 µL |
| ddH_2_O | 11 μL |

Table S2-2. PCR amplification and purification conditions of the target cDNA fragment

| **Temperature** | **Duration** | **Cycles** |
| --- | --- | --- |
| 98 ℃ | 2 min |  |
| 98 ℃ | 10 sec | 40 X |
| 57 ℃ | 30 sec |  |
| 68 ℃ | 2 min |  |
| 68 ℃ | 4 min |  |
| 4 ℃ | ∞ |  |

Table S2-3. Digestion-ligation reaction system

| **Components** | **Volume (15 μL)** |
| --- | --- |
| 10×CutSmart Buffer | 1.5 μL |
| 10 mM ATP | 1.5 μL |
| Empty vector (100 ng/μL) | 1 μL |
| Target fragment (50 ng/μL) | 1 μL |
| *Bsa* I-HF (10 U/μL) | 1 μL |
| T4 DNA ligase (400 U/μL)  dd H_2_O | 1 μL  8μL |

Table S2-4. Digestion-ligation reaction conditions

| **Temperature** | **Duration** | **Cycles** |
| --- | --- | --- |
| 37 ℃ | 5 min | 15 X |
| 20 ℃ | 5 min |  |

Table S2-5. Colony PCR amplification system

| **Components** | **Volume (20 μL)** |
| --- | --- |
| 2× Taq Master MIX | 10 μL |
| 35S-F (10 μM) | 0.5 μL |
| eGFP-cx (10 μM) | 0.5 μL |
| Resuspended colony | 1 μL |
| dd H_2_O | 8 μL |

Table S2-6. Colony PCR amplification conditions

| **Temperature** | **Duration** | **Cycles** |
| --- | --- | --- |
| 95 ℃ | 5 min |  |
| 95 ℃ | 30 sec | 25 X |
| 55 ℃ | 30 sec |  |
| 72 ℃ | 2 min |  |
| 72 ℃ | 4 min |  |
| 4 ℃ | ∞ |  |

Table S2-7. DNA PCR amplification system

| **Components** | **Volume (10μl)** |
| --- | --- |
| 2× Taq Master Mix | 5 μL |
| HygR-F (10 μM) | 0.5 μL |
| HygR-R (10 μM) | 0.5 μL |
| DNA template (100 ng/μL) | 1 μL |
| dd H_2_O | 3 μL |

Table S2-8. DNA PCR amplification conditions

| **Temperature** | **Duration** | **Cycles** |
| --- | --- | --- |
| 95 ℃ | 3 min |  |
| 95 ℃ | 30 sec | 30 X |
| 58 ℃ | 30 sec |  |
| 72 ℃ | 1 min |  |
| 72 ℃ | 5 min |  |
| 4 ℃ | ∞ |  |
