# Supplementary material for: Spermidine improves seed viability in Allium mongolicum by regulating AmCS-mediated metabolic and antioxidant networks
Source: Front Plant Sci. 2025 Oct 8;16:1683362. doi: 10.3389/fpls.2025.1683362 (PMC12540469; doi:10.3389/fpls.2025.1683362)
Supplement: Supplementary file 7 [file Table3.docx]

|  | | Table S3. Statistical analysis of transcriptome quality control data | | | | | | | | |
| --- | --- | --- | --- | --- | --- | --- | --- | --- | --- | --- |
| **Sample** | **Raw Reads** | | **Clean Reads** | **Mapped ratio** | **Uniq mapped ratio** | **Clean Base(G)** | **Error Rate(%)** | **Q20(%)** | **Q30(%)** | **GC(%)** |
| C1-1 | 46842584 | | 44826415 | 85.99% | 18.46% | 6.72 | 0.04 | 98.27 | 94.93 | 45.20 |
| C1-2 | 49950782 | | 47826218 | 85.69% | 18.58% | 7.17 | 0.04 | 98.35 | 95.14 | 45.38 |
| C1-3 | 45390326 | | 43411990 | 83.21% | 18.34% | 6.51 | 0.04 | 98.23 | 94.86 | 44.22 |
| C2-1 | 46747298 | | 44002600 | 82.35% | 21.84% | 6.6 | 0.04 | 97.79 | 94.29 | 46.67 |
| C2-2 | 40745866 | | 39298468 | 82.65% | 21.59% | 5.89 | 0.04 | 98.01 | 94.86 | 46.76 |
| C2-3 | 49621126 | | 48250436 | 82.49% | 22.28% | 7.24 | 0.04 | 98.27 | 95.5 | 45.31 |
| C3-1 | 46487120 | | 44784344 | 81.63% | 21.79% | 6.72 | 0.04 | 97.32 | 93.68 | 44.93 |
| C3-2 | 43383872 | | 41956322 | 82.24% | 21.93% | 6.29 | 0.04 | 97.34 | 93.58 | 45.99 |
| C3-3 | 40265842 | | 39006708 | 82.50% | 21.72% | 5.85 | 0.04 | 97.6 | 94.24 | 45.68 |
| T1-1 | 48911110 | | 46862134 | 85.85% | 18.25% | 7.03 | 0.04 | 98.25 | 94.86 | 45.86 |
| T1-2 | 47008046 | | 44846096 | 86.07% | 18.22% | 6.73 | 0.04 | 98.08 | 94.39 | 46.1 |
| T1-3 | 50057098 | | 47693728 | 85.95% | 18.17% | 7.15 | 0.04 | 97.97 | 94.22 | 45.88 |
| T2-1 | 44290110 | | 42207498 | 80.75% | 19.75% | 6.33 | 0.04 | 97.71 | 94.51 | 45.53 |
| T2-2 | 44709648 | | 42791950 | 81.97% | 19.08% | 6.42 | 0.04 | 97.57 | 94.3 | 45.74 |
| T2-3 | 42846776 | | 40049710 | 78.41% | 18.25% | 6.01 | 0.04 | 97.75 | 94.75 | 45.22 |
| T3-1 | 50460816 | | 48842602 | 85.17% | 20.34% | 7.33 | 0.04 | 98.3 | 95.54 | 46.75 |
| T3-2 | 41248272 | | 39796206 | 83.79% | 20.33% | 5.97 | 0.04 | 97.64 | 93.98 | 47.26 |
| T3-3 | 68381486 | | 64871284 | 82.00% | 21.08% | 9.73 | 0.04 | 97.18 | 92.44 | 44.78 |
