# Supplementary material for: Spermidine improves seed viability in Allium mongolicum by regulating AmCS-mediated metabolic and antioxidant networks
Source: Front Plant Sci. 2025 Oct 8;16:1683362. doi: 10.3389/fpls.2025.1683362 (PMC12540469; doi:10.3389/fpls.2025.1683362)
Supplement: Supplementary file 8 [file Table4.docx]

| Table S4. Statistical analysis of differentially expressed metabolism | | | | | | | |
| --- | --- | --- | --- | --- | --- | --- | --- |
| **Compounds** | **Class** | **T1/C1** | | **T2/C2** | | **T3/C3** | |
|  |  | **VIP** | **FC** | **VIP** | **FC** | **VIP** | **FC** |
| α-D-Glucose 6-phosphate | Phosphate sugars | 1.299 | 1.789 | 1.287 | 1.572 | 0 | 1.774 |
| D-Mannose 6-phosphate | Phosphate sugars | 1.348 | 2.100 | 1.006 | 2.443 | 1.284 | 2.097 |
| dAMP | Nucleotide and its metabolomics | 1.390 | Inf | 1.332 | 2.013 | 0 | 0 |
| Itaconic acid | Organic acid and its derivatives | 1.384 | 1.455 | 2.084 | 1.498 | 1.759 | 1.525 |
| 2,3-Bisphosphoglyceric acid | Phosphoric acids | 1.068 | 0.432 | 1.434 | 1.956 | 0.593 | 1.664 |
| 3-Phenyllactic acid | Organic acid and its derivatives | 0 | 0 | 1.285 | 3.181 | 1.113 | 1.636 |
| Citric acid | Organic acid and its derivatives | 1.009 | 2.209 | 1.297 | 2.243 | 0.998 | 1.687 |
| L-2-Hydroxyglutaric acid disodium salt | Carbohydrate metabolomics | 1.432 | 0 | 1.299 | 2.627 | 1.634 | 2.116 |
| Ornithine | Amino acids | 0 | 1.644 | 1.095 | 3.214 | 2.024 | 1.275 |
| D-Erythrose 4-phosphate | Phosphate sugars | 1.432 | Inf | 1.223 | 1.445 | 1.084 | 1.605 |
| Ureidopropionate | Carbohydrate metabolomics | 1.925 | 2.412 | 1.101 | 1.513 | 0 | 0 |
| Cyclic-AMP | Nucleotide and its metabolomics | 1.408 | 1.260 | 1.053 | 149.7 | 1.743 | 0.483 |
| β-D-Fructose 6-phosphate | Phosphate sugars | 1.456 | 1.664 | 1.370 | 1.772 | 1.838 | 1.543 |
| ATP | Nucleotide and its metabolomics | 1.341 | 2.058 | 1.161 | 1.402 | 1.113 | 3.045 |
| Malic acid | Organic acid and its derivatives | 1.349 | 0.897 | 1.399 | 2.115 | 1.806 | 1.465 |
| β-D-Fructose 1,6-bisphosphate | Phosphate sugars | 1.038 | 0.452 | 1.113 | 0.503 | 1.051 | 0.346 |
| Phosphoenolpyruvate | Organic acid and its derivatives | 1.597 | 1.842 | 1.831 | 1.667 | 1.509 | 1.223 |
| Pyruvate | Organic acid and its derivatives | 1.259 | 1.994 | 1.403 | 1.513 | 1.251 | 1.735 |
| L-Lactate | Organic acid and its derivatives | 1.542 | 1.908 | 1.202 | 1.542 | 1.054 | 1.334 |
| Acetate | Organic acid and its derivatives | 0.233 | 2.583 | 1.053 | 1.363 | 1.023 | 1.487 |
| Acetyl-CoA | Organic acid and its derivatives | 1.877 | 2.216 | 1.196 | 1.753 | 1.673 | 1.558 |
| Glycerone phosphate | Phosphate sugars | 1.118 | 2.186 | 1.615 | 2.662 | 0 | 2.114 |
| D-Ribulose 5-phosphate | Phosphate sugars | 1.336 | 1.987 | 1.042 | 2.046 | 0.557 | 1.035 |
| D-Sedoheptulose 7-phosphate | Phosphate sugars | 0.681 | 1.959 | 1.897 | 1.226 | 1.688 | 1.886 |

Variable Importance in Projection (VIP) scores (>1.0) reflect the significance of metabolites in discriminating sample groups (e.g., in OPLS-DA models). Metabolites with a fold change (FC) ≥1.5 or ≤0.5 (experimental vs. control) were considered significantly altered.
