# Supplementary material for: Spermidine improves seed viability in Allium mongolicum by regulating AmCS-mediated metabolic and antioxidant networks
Source: Front Plant Sci. 2025 Oct 8;16:1683362. doi: 10.3389/fpls.2025.1683362 (PMC12540469; doi:10.3389/fpls.2025.1683362)
Supplement: Supplementary file 9 [file Table5.docx]

| Table S5. Screening of energy metabolism genes | | | | | | |
| --- | --- | --- | --- | --- | --- | --- |
| **Gene ID** | **C1** | **C2** | **C3** | **T1** | **T2** | **T3** |
| **Hexokinase (*HK*)** | | | | | | |
| Cluster-103494.7 | 3.243 | 2.296 | 0.595 | 3.746 | 2.093 | 2.356 |
| Cluster-103494.8 | 51.135 | 24.133 | 1.446 | 50.257 | 29.713 | 18.786 |
| Cluster-52388.1 | 7.212 | 4.837 | 0.827 | 8.286 | 6.434 | 3.765 |
| Cluster-108424.3 | 6.336 | 3.837 | 3.272 | 14.219 | 8.249 | 9.092 |
| Cluster-63250.0 | 8.114 | 6.417 | 0.013 | 12.089 | 7.487 | 3.367 |
| Cluster-88662.1 | 4.099 | 2.776 | 0.462 | 11.097 | 7.073 | 2.097 |
| Cluster-98424.0 | 4.829 | 2.057 | 2.933 | 62.913 | 127.433 | 2.377 |
| **Glucose-6-phosphate isomerase (*GPI*)** | | | | | | |
| Cluster-107916.8 | 0.396 | 0.163 | 0.028 | 1.244 | 0.374 | 0.419 |
| Cluster-15057.0 | 0.287 | 0.053 | 0.012 | 2.273 | 0.761 | 0.319 |
| **Phosphofructokinase (*PFK9*)** | | | | | | |
| Cluster-32915.1 | 76.526 | 53.376 | 8.756 | 83.463 | 44.393 | 35.986 |
| Cluster-81383.1 | 31.847 | 9.557 | 0.463 | 37.246 | 18.286 | 5.987 |
| Cluster-81383.2 | 66.113 | 37.253 | 19.426 | 77.063 | 51.683 | 31.156 |
| Cluster-96534.0 | 9.086 | 5.766 | 0.157 | 10.163 | 5.623 | 2.949 |
| Cluster-45682.2 | 18.163 | 12.437 | 3.007 | 27.129 | 16.104 | 8.126 |
| **Pyrophosphate-dependent phosphofructokinase (*PFP*)** | | | | | | |
| Cluster-115744.0 | 86.093 | 37.027 | 4.087 | 90.467 | 41.753 | 23.627 |
| Cluster-22102.0 | 3.197 | 1.117 | 0.623 | 3.977 | 1.707 | 1.015 |
| Cluster-78007.3 | 4.283 | 3.966 | 1.017 | 4.497 | 4.423 | 3.143 |
| Cluster-98659.1 | 3.307 | 1.543 | 0.083 | 3.763 | 1.917 | 1.406 |
| **Fructose-1,6-bisphosphatase (*FBP*)** | | | | | | |
| Cluster-100141.2 | 16.487 | 1.353 | 0.943 | 19.413 | 4.777 | 1.087 |
| Cluster114595.14 | 164.773 | 45.103 | 22.443 | 168.073 | 70.843 | 42.687 |
| Cluster-93304.1 | 5.163 | 3.717 | 1.703 | 6.917 | 4.553 | 1.707 |
| **Triosephosphate isomerase (*TPI*)** | | | | | | |
| Cluster-19883.0 | 2.267 | 1.033 | 0.727 | 4.257 | 0.000 | 3.981 |
| Cluster-95551.1 | 71.197 | 32.877 | 21.263 | 81.847 | 40.033 | 35.967 |
| **Fructose-bisphosphate aldolase (*ALDO*)** | | | | | | |
| Cluster-120950.1 | 85.333 | 47.723 | 4.493 | 98.737 | 68.607 | 27.413 |
| Cluster-120950.4 | 1801.043 | 1167.373 | 1001.426 | 2421.350 | 1811.497 | 1038.583 |
| Cluster-120950.2 | 627.623 | 372.973 | 62.973 | 763.437 | 471.727 | 634.187 |
| Cluster-120950.5 | 1132.427 | 859.597 | 38.547 | 1318.793 | 782.987 | 483.053 |
| Cluster-95043.1 | 16.237 | 7.723 | 6.087 | 22.737 | 10.607 | 12.893 |
| Cluster-118998.1 | 9.223 | 3.983 | 1.603 | 8.617 | 4.143 | 2.767 |
| Cluster-82935.0 | 3.303 | 0.167 | 0.118 | 13.843 | 1.453 | 0.537 |
| Cluster-42507.3 | 1020.183 | 780.167 | 149.943 | 769.407 | 528.261 | 53.392 |
| Cluster-99736.0 | 4.023 | 1.943 | 1.003 | 11.139 | 4.227 | 3.943 |
| **Glyceraldehyde-3-phosphate dehydrogenase (*GAPDH*)** | | | | | | |
| Cluster-82675.0 | 1.453 | 0.502 | 0.182 | 4.427 | 0.717 | 0.313 |
| Cluster-89930.0 | 6.473 | 0.067 | 0.043 | 32.147 | 2.307 | 0.203 |
| Cluster-66445.1 | 22.907 | 2.307 | 1.203 | 21.020 | 5.117 | 4.017 |
| **Phosphoglycerate kinase (*PGK*)** | | | | | | |
| Cluster-91816.0 | 1.427 | 0.143 | 0.073 | 4.607 | 0.453 | 0.382 |
| **NADP+dependent glyceraldehyde-3-phosphate dehydrogenase (*gapN*)** | | | | | | |
| Cluster-96870 | 42.513 | 25.523 | 3.727 | 48.467 | 36.964 | 12.033 |
| **Phosphoglycerate mutase (*PGAM*)** | | | | | | |
| Cluster-111990.0 | 8.437 | 3.377 | 2.154 | 10.427 | 4.447 | 4.017 |
| Cluster-118395.1 | 46.457 | 37.467 | 8.363 | 79.137 | 42.313 | 41.257 |
| Cluster-91234.0 | 9.193 | 8.143 | 0.863 | 12.743 | 8.667 | 7.593 |
| **2,3-bisphosphoglycerate-independent phosphoglycerate mutase (*gpmI*)** | | | | | | |
| Cluster-110156.9 | 14.193 | 3.533 | 0.747 | 18.857 | 9.447 | 2.723 |
| Cluster-60396.7 | 148.707 | 52.587 | 1.897 | 155.093 | 69.687 | 27.243 |
| Cluster-60396.8 | 207.813 | 99.857 | 14.533 | 209.784 | 141.373 | 43.656 |
| **Multiple inositol polyphosphate phosphatase 1 (*MINPP1*)** | | | | | | |
| Cluster-82343.3 | 24.203 | 6.047 | 3.123 | 21.407 | 7.463 | 7.873 |
| Cluster-82343.4 | 3.363 | 2.843 | 1.883 | 10.103 | 8.893 | 3.373 |
| **Pyruvate phosphate dikinase (*ppdK*)** | | | | | | |
| Cluster-99739.0 | 36.707 | 4.627 | 4.127 | 34.643 | 9.797 | 5.877 |
| **Enolase 1 (*ENO1*)** | | | | | | |
| Cluster-76089.0 | 2.363 | 0.013 | 0.000 | 13.557 | 1.003 | 0.333 |
| Cluster-115469.2 | 5.343 | 0.177 | 0.027 | 5.407 | 2.023 | 1.733 |
| Cluster-115469.3 | 2.353 | 0.000 | 1.113 | 8.323 | 9.183 | 1.677 |
| Cluster-115469.5 | 11.823 | 9.127 | 2.353 | 18.847 | 15.178 | 8.218 |
| **Pyruvate Kinase (*PK*)** | | | | | | |
| Cluster-117021.1 | 92.323 | 84.407 | 61.157 | 99.317 | 92.003 | 68.353 |
| Cluster-117021.6 | 0.983 | 0.637 | 0.313 | 1.673 | 1.653 | 0.367 |
| Cluster-22771.0 | 0.863 | 0.147 | 0.000 | 1.113 | 2.033 | 2.463 |
| Cluster-90032.0 | 168.487 | 86.167 | 2.913 | 198.623 | 102.253 | 77.557 |
| Cluster-90032.17 | 10.487 | 1.373 | 0.000 | 14.857 | 3.363 | 8.073 |
| Cluster-90032.6 | 6.483 | 2.513 | 0.000 | 9.203 | 6.293 | 4.833 |
| Cluster-93046.14 | 3.313 | 0.747 | 0.317 | 3.733 | 4.007 | 2.013 |
| Cluster-93046.2 | 3.287 | 1.292 | 0.427 | 4.713 | 2.287 | 0.497 |
| Cluster-93046.5 | 0.853 | 0.053 | 0.223 | 1.713 | 0.733 | 0.853 |
| Cluster-96010.0 | 17.603 | 13.013 | 4.263 | 16.470 | 16.350 | 8.330 |
| Cluster-106853.3 | 3.187 | 1.963 | 1.063 | 7.253 | 3.198 | 4.367 |
| Cluster-115341.0 | 3.053 | 0.393 | 0.203 | 8.267 | 1.183 | 0.407 |
| Cluster-31143.0 | 2.017 | 1.593 | 0.557 | 3.602 | 2.022 | 1.133 |
| Cluster-36465.0 | 66.073 | 32.007 | 11.128 | 95.271 | 41.192 | 17.198 |
| Cluster-86858.0 | 1.763 | 0.183 | 0.113 | 10.273 | 1.123 | 1.817 |
| **Lactate Dehydrogenase (*LDH*)** | | | | | | |
| Cluster-2184.0 | 2.177 | 1.892 | 1.312 | 4.116 | 3.273 | 3.943 |
| **Citrate Synthase (*CS*)** | | | | | | |
| Cluster-115292.3 | 2.413 | 1.497 | 1.647 | 3.207 | 1.527 | 1.813 |
| Cluster-121661.3 | 9.357 | 7.430 | 4.631 | 8.150 | 4.220 | 2.793 |
| Cluster-121661.6 | 16.187 | 11.050 | 8.842 | 18.730 | 12.427 | 10.730 |
| Cluster-60376.0 | 0.133 | 0.093 | 0.046 | 0.880 | 1.733 | 0.120 |
| Cluster-94953.0 | 194.247 | 123.033 | 89.971 | 203.137 | 138.377 | 102.243 |
| Cluster-98505.0 | 182.403 | 147.227 | 98.863 | 190.287 | 153.957 | 99.367 |
| Cluster-80299.0 | 0.947 | 1.473 | 2.463 | 0.743 | 0.137 | 0.423 |
| **1-Aminocyclopropane-1-carboxylate oxidase (*ACO*)** | | | | | | |
| Cluster-104838.2 | 111.547 | 81.933 | 77.753 | 128.317 | 107.933 | 139.393 |
| Cluster-117774.2 | 13.267 | 12.183 | 11.637 | 14.153 | 15.883 | 12.533 |
| Cluster-117774.6 | 78.727 | 51.057 | 46.983 | 75.357 | 53.507 | 59.863 |
| Cluster-64147.0 | 1.067 | 0.601 | 0.000 | 4.413 | 2.597 | 0.067 |
| **Isocitrate Dehydrogenase (*IDH1*, *IDH2*)** | | | | | | |
| Cluster-92180.0 | 12.937 | 4.383 | 7.743 | 12.953 | 8.507 | 8.473 |
| Cluster-92509.0 | 173.053 | 4.083 | 54.273 | 158.973 | 32.367 | 51.347 |
| **Oxoglutarate Dehydrogenase (*OGDH*)** | | | | | | |
| Cluster-101678.0 | 11.573 | 8.933 | 4.283 | 43.073 | 15.377 | 20.087 |
| Cluster-21328.0 | 8.643 | 4.073 | 0.000 | 9.057 | 5.673 | 0.277 |
| Cluster-27511.0 | 6.863 | 0.213 | 0.023 | 9.627 | 1.533 | 1.725 |
| Cluster-91842.0 | 35.953 | 22.283 | 22.417 | 40.973 | 28.717 | 26.913 |
| Cluster-101678.5 | 0.000 | 25.973 | 12.235 | 0.000 | 5.833 | 3.753 |
| **Succinyl-CoA ligase [ADP-forming] subunit alpha (*sucD*)** | | | | | | |
| Cluster-39541.2 | 36.633 | 32.697 | 29.027 | 38.023 | 39.293 | 28.743 |
| Cluster-45921.0 | 1.085 | 0.297 | 0.000 | 1.329 | 2.143 | 0.000 |
| Cluster-22567.0 | 0.763 | 0.187 | 0.215 | 3.927 | 2.267 | 0.263 |
| Cluster-94657.2 | 59.323 | 43.743 | 33.493 | 52.007 | 46.723 | 35.743 |
| Cluster-86803.0 | 5.713 | 3.407 | 0.473 | 6.253 | 4.253 | 0.483 |
| Cluster-47335.0 | 6.723 | 5.357 | 1.267 | 3.263 | 0.537 | 0.247 |
| **Succinate dehydrogenase [ubiquinone] flavoprotein subunit (*SDHA*)** | | | | | | |
| Cluster-106832.0 | 61.677 | 55.547 | 47.237 | 79.043 | 64.413 | 56.683 |
| Cluster-106832.1 | 33.027 | 20.367 | 18.863 | 33.057 | 26.517 | 19.887 |
| Cluster-31829.0 | 1.593 | 0.557 | 0.000 | 2.187 | 4.883 | 2.377 |
| Cluster-102430.0 | 34.653 | 28.913 | 25.057 | 24.307 | 17.407 | 16.533 |
| Cluster-122024.2 | 4.297 | 2.837 | 0.263 | 5.017 | 3.263 | 0.763 |
| **Fumarate hydratase (f*umA*)** | | | | | | |
| Cluster-46389.0 | 2.277 | 1.193 | 1.283 | 3.717 | 2.013 | 2.623 |
| **Malate Dehydrogenase (*MDH1*)** | | | | | | |
| Cluster-13832.0 | 0.000 | 0.027 | 0.000 | 0.000 | 3.763 | 0.000 |
| Cluster-24241.0 | 4.273 | 1.343 | 1.155 | 7.187 | 5.963 | 0.000 |
| Cluster-91740.0 | 120.173 | 114.267 | 79.873 | 131.163 | 99.827 | 89.337 |
| Cluster-119458.1 | 20.497 | 11.373 | 0.323 | 0.463 | 0.893 | 1.333 |
| Cluster-27659.0 | 6.217 | 10.133 | 11.127 | 7.003 | 1.383 | 0.719 |
| Cluster-99125.1 | 4.593 | 2.623 | 1.407 | 5.733 | 3.507 | 2.183 |
| **Dihydrolipoamide S-succinyltransferase (*DLST*)** | | | | | | |
| Cluster-107664.0 | 131.767 | 63.213 | 7.837 | 163.737 | 71.247 | 47.847 |
| Cluster-107664.2 | 2.393 | 1.867 | 0.783 | 2.537 | 2.653 | 1.173 |
| Cluster-107664.4 | 5.877 | 4.317 | 0.277 | 7.603 | 4.593 | 4.353 |
| Cluster-107664.6 | 21.333 | 16.283 | 3.393 | 21.547 | 12.993 | 0.000 |
| Cluster-121790.3 | 57.043 | 33.503 | 2.787 | 73.233 | 34.013 | 15.123 |
| Cluster-18847.0 | 57.043 | 33.503 | 2.787 | 103.233 | 64.013 | 8.887 |
| **Glucose-6-phosphate dehydrogenase (*G6PD*)** | | | | | | |
| Cluster-103561.1 | 4.163 | 1.683 | 0.233 | 5.083 | 2.136 | 1.573 |
| Cluster-103561.7 | 7.547 | 4.760 | 2.903 | 7.123 | 5.413 | 5.377 |
| Cluster-108992.0 | 3.233 | 1.883 | 0.000 | 19.167 | 13.103 | 1.077 |
| Cluster-34698.0 | 4.943 | 2.657 | 0.257 | 0.000 | 3.703 | 1.563 |
| Cluster-85321.0 | 36.787 | 22.543 | 0.087 | 4.543 | 3.633 | 0.053 |
| **6-Phosphogluconolactonase (*PGLS*)** | | | | | | |
| Cluster-100066.7 | 17.507 | 13.523 | 5.967 | 78.947 | 54.113 | 68.667 |
| **Phosphogluconate dehydrogenase (*PGD*)** | | | | | | |
| Cluster-117113.0 | 23.543 | 5.517 | 0.353 | 43.223 | 11.163 | 3.187 |
| Cluster-87596.0 | 29.537 | 15.447 | 12.123 | 30.923 | 19.893 | 14.063 |
| Cluster-93211.0 | 111.493 | 57.273 | 13.937 | 110.823 | 76.873 | 34.243 |
| **Ribose-5-phosphate isomerase A (*rpiA*)** | | |  |  |  |  |
| Cluster-106198.0 | 50.213 | 40.837 | 4.823 | 49.787 | 44.183 | 12.953 |
| Cluster-115623.0 | 7.897 | 6.993 | 0.877 | 8.777 | 6.377 | 3.423 |
| ,Cluster-117530.1 | 35.753 | 16.597 | 10.753 | 33.033 | 21.643 | 19.443 |
| Cluster-71294.0 | 29.677 | 27.127 | 4.313 | 28.913 | 28.567 | 12.277 |
| Cluster-98111.0 | 16.543 | 14.023 | 7.113 | 20.133 | 14.833 | 11.067 |
| Cluster-46348.1 | 3.187 | 3.057 | 0.293 | 4.723 | 3.113 | 0.893 |
| **Ribulose-5-phosphate 3-epimerase (*RPE*)** | | | | | | |
| Cluster-110414.0 | 65.053 | 49.377 | 28.087 | 57.243 | 52.557 | 45.393 |
| Cluster-97547.1 | 22.963 | 7.657 | 5.617 | 29.723 | 11.443 | 7.223 |
| Cluster-91346.0 | 11.173 | 10.533 | 0.133 | 5.153 | 2.853 | 0.173 |
| **Transketolase (*tktA*)** | | | | | | |
| Cluster-114267.0 | 29.127 | 12.457 | 1.187 | 28.433 | 22.247 | 11.456 |
| Cluster-118911.1 | 114.427 | 69.537 | 5.343 | 129.154 | 87.935 | 31.503 |
| Cluster-113468.0 | 2.473 | 1.317 | 0.003 | 2.863 | 0.000 | 0.143 |
| Cluster-118911.0 | 14.576 | 0.147 | 0.063 | 12.167 | 0.387 | 0.047 |
| **Transaldolase B (*talB*)** | | | | | | |
| Cluster-111820.1 | 97.694 | 34.463 | 2.766 | 108.257 | 43.134 | 9.464 |
| Cluster-111820.2 | 152.845 | 125.073 | 78.853 | 143.635 | 126.393 | 105.537 |
| Cluster-32319.1 | 2.503 | 134.147 | 136.336 | 2.926 | 42.057 | 60.875 |
| **Phosphoribosyl pyrophosphate synthetase (*PRPS*)** | | | | | | |
| Cluster-7126.0 | 61.003 | 17.467 | 5.768 | 85.317 | 40.444 | 47.393 |
| **Pyruvate Decarboxylase (*PDC*)** | | | | | | |
| Cluster-7063.0 | 12.945 | 8.513 | 0.193 | 21.867 | 21.718 | 4.406 |
| Cluster-122083.0 | 1.267 | 0.757 | 0.653 | 1.763 | 1.513 | 1.387 |
| **Acetyl-CoA Synthetase (*ACSS*)** | | | | | | |
| Cluster-89717.0 | 25.947 | 23.473 | 11.252 | 33.357 | 27.523 | 15.196 |
| Cluster-92399.1 | 22.981 | 13.956 | 11.210 | 23.723 | 16.363 | 18.797 |
| Cluster-94339.0 | 25.485 | 23.681 | 13.617 | 27.037 | 26.237 | 19.623 |
| Cluster-104544.2 | 12.437 | 7.696 | 4.896 | 15.353 | 10.916 | 6.493 |
| Cluster-104544.3 | 11.053 | 7.133 | 5.666 | 13.113 | 9.823 | 6.567 |
| Cluster-91932.0 | 69.049 | 51.306 | 42.488 | 73.854 | 63.307 | 51.703 |
| **Chalcone Synthase (*CHS*)** | | | | | | |
| Cluster-33671.1 | 4.523 | 2.533 | 1.840 | 6.864 | 7.324 | 7.543 |
| Cluster-40727.0 | 2.543 | 1.667 | 1.213 | 4.643 | 3.953 | 2.973 |
| [**Chalcone isomerase (*CHI*)**](https://www.kegg.jp/entry/5.5.1.6) | | | | | | |
| Cluster-121260.0 | 5.713 | 7.197 | 8.417 | 4.793 | 2.227 | 4.923 |
| Cluster-33373.0 | 0.433 | 0.267 | 0.153 | 2.507 | 1.693 | 1.283 |
| Cluster-62941.4 | 1.077 | 2.097 | 2.117 | 27.453 | 31.437 | 21.297 |
| Cluster-82884.6 | 228.557 | 202.893 | 189.57 | 19.493 | 20.477 | 12.393 |
| Cluster-48145.1 | 1.597 | 1.297 | 1.023 | 6.573 | 7.095 | 5.347 |
| Cluster-48145.4 | 6.753 | 3.197 | 1.733 | 11.223 | 8.193 | 5.527 |
| **Flavonoid 3',5'-hydroxylase (*CYP75A*)** | | | | | | |
| Cluster-105103.7 | 2.153 | 1.123 | 1.007 | 3.893 | 3.883 | 2.857 |
| Cluster-105103.9 | 15.093 | 9.177 | 2.523 | 17.823 | 21.493 | 16.433 |
| **Flavonoid 3'-hydroxylase (CYP75B1)** | | | | | | |
| Cluster-95420.0 | 2.843 | 1.760 | 0.433 | 4.603 | 4.867 | 2.383 |
| Cluster-76646.0 | 4.923 | 5.350 | 9.740 | 2.623 | 1.517 | 1.073 |
| **Dihydroflavonol 4-reductase (*DFR*)** | | | | | | |
| Cluster-116873.2 | 19.863 | 17.027 | 13.973 | 31.533 | 21.393 | 17.877 |
| Cluster-120382.1 | 17.397 | 22.843 | 28.873 | 14.013 | 12.253 | 17.193 |
| **Flavanone 7-O-glucoside 2''-O-beta-L-rhamnosyltransferase (*C12RT1*)** | | | | | | |
| Cluster-62076.1 | 7.973 | 6.603 | 4.657 | 35.207 | 30.097 | 17.553 |
| Cluster-62076.10 | 5.983 | 6.187 | 8.557 | 4.473 | 4.573 | 6.223 |
| Cluster-62076.14 | 51.720 | 41.050 | 32.210 | 76.483 | 62.757 | 44.367 |
| Cluster-62076.2 | 7.273 | 4.957 | 2.543 | 27.093 | 15.647 | 9.887 |
| Cluster-62076.4 | 129.667 | 126.027 | 106.803 | 14.677 | 13.573 | 12.773 |
| Cluster-62076.7 | 5.427 | 3.853 | 2.833 | 12.343 | 7.743 | 4.583 |
| **Flavonol Synthase (*FLS*)** | | | | | | |
| Cluster-118629.3 | 1.457 | 1.383 | 1.120 | 7.283 | 5.877 | 2.223 |
| Cluster-118629.6 | 9.293 | 1.377 | 0.853 | 13.443 | 3.353 | 1.667 |
| Cluster-69586.0 | 349.370 | 356.390 | 483.31 | 134.667 | 135.627 | 189.363 |
| **Hydroxycinnamoyl-CoA Shikimate/Quinate Hydroxycinnamoyltransferase (*HCT*)** | | | | | | |
| Cluster-100335.0 | 10.323 | 11.577 | 20.933 | 7.877 | 6.357 | 8.757 |
| Cluster-111776.0 | 14.093 | 8.913 | 6.487 | 56.447 | 37.127 | 31.877 |
| Cluster-111776.4 | 11.317 | 9.873 | 2.397 | 23.477 | 16.133 | 4.777 |
| Cluster-111776.5 | 16.713 | 6.577 | 2.133 | 26.893 | 9.253 | 7.567 |
| Cluster-114626.0 | 2.023 | 1.133 | 0.913 | 13.013 | 2.733 | 1.303 |
| Cluster-116502.1 | 2.467 | 2.413 | 1.973 | 11.763 | 12.403 | 6.403 |
| Cluster-56778.2 | 0.317 | 0.237 | 0.213 | 4.573 | 4.423 | 2.563 |
| **Anthocyanidin Reductase (*AN*R)** | | | | | | |
| Cluster-19373.0 | 9.323 | 7.113 | 2.373 | 17.713 | 9.443 | 4.023 |
| **Leucoanthocyanidin Reductase (*LAR*)** | | | | | | |
| Cluster-90993.0 | 24.223 | 6.813 | 0.317 | 7.233 | 6.857 | 3.093 |
| **4-Hydroxyphenylpyruvate Reductase (*HPPR*)** | | | | | | |
| Cluster-53522.0 | 24.033 | 21.543 | 8.627 | 28.053 | 28.223 | 14.567 |
