# Supplementary material for: Spermidine improves seed viability in Allium mongolicum by regulating AmCS-mediated metabolic and antioxidant networks
Source: Front Plant Sci. 2025 Oct 8;16:1683362. doi: 10.3389/fpls.2025.1683362 (PMC12540469; doi:10.3389/fpls.2025.1683362)
Supplement: Supplementary file 10 [file Table6.docx]

| Table S6. Critical values of η² for significance in one-way ANOVA | | | | | |
| --- | --- | --- | --- | --- | --- |
| **Figure Number** | **η²** | **P** | **Figure Number** | **η²** | **P** |
| Figure S1A | 0.7654 | 0.0100 | Figure S1B | 0.8124 | 0.0315 |
| Figure 3D-HK | 0.8765 | 0.0012 | Figure 3E-CS | 0.4567 | 0.0101 |
| Figure 3D-PK | 0.4432 | 0.0122 | Figure 3E-IDH | 0.4248 | 0.0372 |
| Figure 3D-PHK | 0.1123 | 0.0342 | Figure 3E-OGDH | 0.6931 | 0.0128 |
| Figure 3D-ATP | 0.7876 | 0.0121 | Figure 3E-MDH | 0.6452 | 0.0431 |
| Figure 3F | 0.8321 | 0.0017 | Figure 4I | 0.8723 | 0.0085 |
| Figure 5C-GR | 0.7456 | 0.0091 | Figure 5C-GE | 0.3376 | 0.0214 |
| Figure 5E-SOD | 0.2987 | 0.0016 | Figure 5F-H_2_O_2_ | 0.7815 | 0.0199 |
| Figure 5E-POD | 0.1345 | 0.0412 | Figure 5F-DMA | 0.8249 | 0.0037 |
| Figure 5E-CAT | 0.5112 | 0.0013 | Figure 5G-PDC | 0.2580 | 0.0176 |
| Figure 5E-APX | 0.6567 | 0.0064 | Figure 5G- AmCS | 0.8012 | 0.0293 |
| Figure 5G- CS | 0.7723 | 0.0082 | Figure 5G- G6PD | 0.4023 | 0.0012 |
| Figure 5G- IDH | 0.4234 | 0.0376 | Figure 5G-HK | 0.5645 | 0.0247 |
| Figure 5G- OGDH | 0.5654 | 0.0092 | Figure 5G-PK | 0.4158 | 0.0093 |
| Figure 5G- MDH | 0.2789 | 0.0075 | Figure 5G-PFK | 0.6376 | 0.0384 |
| Figure 5G-ATP | 0.8845 | 0.0013 | Figure 5G-NADPH | 0.6734 | 0.0162 |
| Figure 5G- Respiratory Rate | 0.2098 | 0.0042 | Figure 5G- acetyl-CoA | 0.8881 | 0.0471 |

This table summarizes the effect sizes (η², eta-squared) and p-values from one-way ANOVA analyses of the data presented in the indicated figures. η² represents the proportion of the total variance attributable to the experimental treatment factor. All statistically significant effects reported in the main text are listed herein. The significance level (α) was set at 0.05 for all analyses. Data are presented as mean ± SD (n = 3).
